# Taming Fabry–Pérot resonances in a dual-metasurface multiband antenna with beam steering in one of the bands

**DOI:** 10.1038/s41598-023-36828-4

**Published:** 2023-06-19

**Authors:** Rafael Gonçalves Licursi de Mello, Anne Claire Lepage, Xavier Begaud

**Affiliations:** Laboratoire de Traitement et Communication de l′Information (LTCI), Télécom Paris, Institut Polytechnique de Paris, 91120 Palaiseau, France

**Keywords:** Electrical and electronic engineering, Applied physics

## Abstract

Metasurfaces are artificial materials that can provide properties not readily available in nature for the interaction with acoustic, elastodynamic, or electromagnetic waves. In Electromagnetics, metasurfaces allow particular functionalities to antennas, which in turn lately have been increasingly pushed to a multiband operation. To fully exploit metasurfaces’ capabilities, the use of a metasurface reflector and a metasurface superstrate surrounding a radiating element in multiband antennas is interesting. However, such topology generally creates multiple reflections inside the formed cavity, known as Fabry–Pérot resonances. Here we show that one should tame this phenomenon to use two parallel metasurfaces surrounding a planar radiating element. We present the conditions to obtain directive, multiband antennas under such circumstances. The concepts are validated with a compact device for 5G/4G/Wi-Fi 2.4/5/6E performing a beam steering in the 5G without disturbing the radiation patterns of the other bands. This device demonstrates that the functionalities of two metasurfaces may be exploited in a single design if the presented conditions are respected. We also anticipate our work to be a starting point for other studies in the wave domain. For example, compact, multiband, beam-steerable microphones or sonar transducers with two parallel metasurfaces could be investigated in the future.

## Introduction

The scientific community witnessed in the last decades a remarkable evolution of artificial engineered materials that can be used with traditional components to obtain particular functionalities. Metasurfaces interact with waves in an exotic manner providing properties not readily found in natural materials used in Acoustics^1,2^, Elastodynamics^3,4^, Electromagnetics, among others. In Electromagnetics, they are used to absorb^5,6^ or control the polarization^7,8^ of waves. Metasurfaces also allow an asymmetric transmission^9^, imposing different effects on the waves depending on the direction they are traversed. Moreover, these structures can steer^10–14^, focus^14,15^, and even shape^13–18^ beams of electromagnetic waves. Metasurfaces can also work as artificial magnetic conductors (AMC), reflecting waves without phase shift at a single^19^ or multiple frequencies^20^, which can lead to low-profile devices^21^ and multiband reflection schemes^22^. The steering of beams is particularly interesting because it allows not only the optimization of radio links at specific directions, but also facilitates the measurement of the electromagnetic properties of samples under test^23,24^.

**Figure 1:**
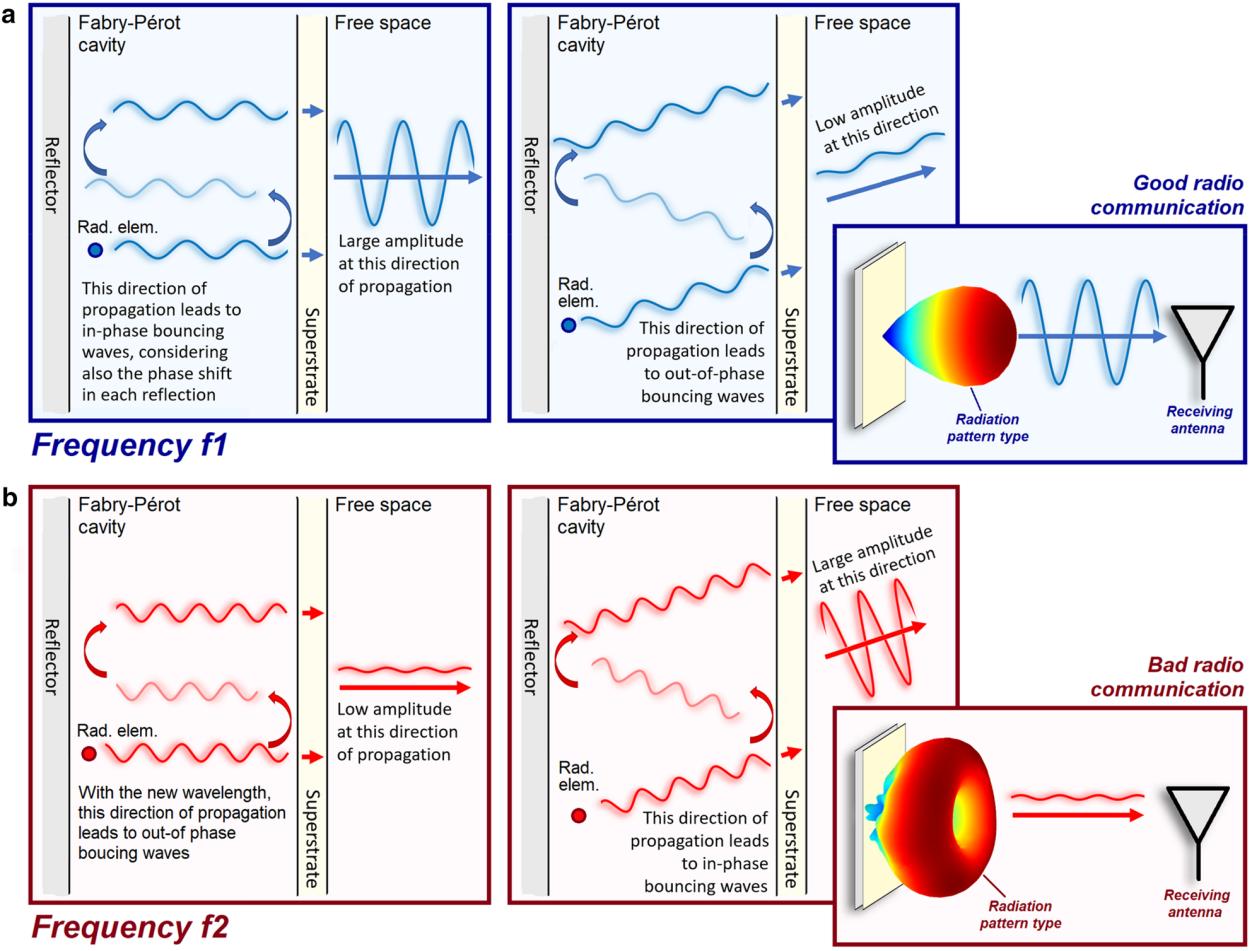
Outside the Fabry–Pérot cavity, waves interfere either constructively or destructively at each angle depending on the path travelled inside the cavity and the phase shift due to reflections on the reflector and the superstrate. (**a**) Constructive interferences occur at the broadside direction at the frequency $f=f1.$ (**b**) Destructive interferences occur at the broadside direction at the frequency $f=f2$.

The above functionalities allow the glimpse of a whole plethora of new technologies that can highly impact the performance of numerous devices. If two metasurfaces are used the degrees of freedom are even higher. One example is the beam-steerable antenna^25^ made of a dipole radiating element surrounded by a partially reflective metasurface as a superstrate and an AMC reflector. When the dipole radiates, some energy is transmitted through the superstrate. The rest of the energy is reflected, bouncing inside the cavity formed by the metasurfaces and impinging again upon the superstrate. Then, another portion of energy is transmitted, and the cycle repeats in what is called Fabry–Pérot resonances^26^. Outside the cavity, interference patterns result in one or more beams with specific widths and directions depending on the superstrate properties and the phase shift between subsequent bouncing waves. Figure 1a depicts Fabry–Pérot resonances for a case where constructive interferences occur at the broadside direction, while destructive interferences occur at other directions, leading to a radiation pattern that prioritizes radio communication with other antennas at the broadside direction. In that work^25^, the combined functionalities consisted of the AMC (which controls the phase shift of the waves it reflects) steering a narrow and high-gain beam enabled by the superstrate.

In the above work^25^, the authors intended to design a Fabry–Pérot cavity. Nevertheless, Fabry–Pérot resonances should be carefully handled whenever an antenna presents a non-perfectly transparent superstrate parallel to a reflector, at risk of creating spurious interference patterns outside the cavity, deforming, or deflecting the beams to undesired directions. A recent work^27^ used two parallel metasurfaces surrounding a radiating element to generate independent beams with polarization conversion at $12.0$ and $15.0\,\mathrm{GHz}$. Even if such device was not intended to be a Fabry–Pérot cavity, its superstrate was not perfectly transparent. However, by separating the two metasurfaces with a distance that led to constructive interferences at the broadside direction for both operating frequencies, the Fabry–Pérot resonances were tamed. This was possible because the ratio between the highest and lowest operating frequencies was close to one (${f}_{ratio}=1.25$).

If the frequency ratio is not close to one, taming Fabry–Pérot resonances becomes a challenge, because the wavelength considerably varies from a band to another thereby changing the interference patterns, whether the device is intended to be a Fabry–Pérot cavity or not. Figure 1b illustrates a case where due to a significant change in the wavelength destructive interferences started to occur at the broadside direction, whereas constructive interferences started to happen at other directions, leading to a radiation pattern with a null in the broadside direction. In this case, a radio link established in one band may severely deteriorate in another. Another recent work^28^ proposed a triple-band antenna made by an outer radiating element (operating from $1.71$ to $2.69\,\mathrm{GHz}$) covering an inner, dual-band radiating element (for the ranges $3.30$–$3.60$ and $4.80$–$5.00\,\mathrm{GHz}$), both backed by a common reflector. The outer element worked as a superstrate for the inner one, creating reflections and an unintentional Fabry–Pérot cavity for the two upper bands. The frequency ratio related to the two upper bands was not close to one (${f}_{ratio}=1.52$). Resonating elements were used to minimize the reflections on the superstrate (the outer element) and obtain a good performance. Despite a reflection coefficient magnitude lower than $-10\,\mathrm{dB}$, the Fabry–Pérot resonances were still relevant, changing the radiation patterns for the upper band depending on whether the superstrate was present or not.

The above works show that if not properly tamed Fabry–Pérot resonances can be a barrier to a multiband operation of a radiating element surrounded by two metasurfaces. Here we present the conditions for a directive, multiband operation in such topology despite the presence of undesired Fabry–Pérot resonances. The concepts are illustrated with an antenna for the 5G/4G/Wi-Fi 2.4/5/6E standards, operating in the frequency ranges $2.40$–$2.70$, $3.40{-}3.80$, $5.17$–$5.83$, and $5.93$–$6.45\,\mathrm{GHz}$ (hereafter called B1–B4 bands), which leads to a frequency ratio ${f}_{ratio}=2.69$. A reconfigurable Huygens metasurface allows a beam-steering in the 5G without disturbing the radiation patterns of the other standards. A dual-band AMC, besides providing low-profile features, works as a key element to tame the Fabry–Pérot resonances. Since such resonances are common to the wave domain, we believe this work can be a starting point for studies in other areas, such as Acoustics, and Elastodynamics, e.g., compact, multiband, beam-steerable microphones or sonar transducers using two parallel metasurfaces could be investigated in the future.

## Results

### Conditions for stable, directive radiation pattern

The condition to enhance the gain at the broadside direction of a planar antenna spaced a distance ${h}_{refl}$ from a parallel reflector was recently presented^29^. The condition to obtain optimal, constructive interferences at the broadside direction of an antenna presenting a superstrate parallel to a reflector, both with reflection coefficients ${\Gamma }_{sup}$ and ${\Gamma }_{refl}$ and separated by a distance ${h}_{FP}$, was stated some decades ago^30^. Since the radiating element is between the reflector and the superstrate in the topology in question, we identify that three conditions are essential to keep a stable, directive radiation pattern with a maximum gain in the broadside direction:

1. The reflector-backed antenna broadside gain enhancement condition^29^:

1 $$-120^\circ +2k{h}_{refl}+2N\pi <{\varphi }_{{\Gamma }_{refl}}<+120^\circ +2k{h}_{refl}+2N\pi$$

where $N\in {\mathbb{Z}}$, ${\varphi }_{{\Gamma }_{refl}}$ is the reflection coefficient phase of the reflector, $k=2\pi f/c$ is the wavenumber, $f$ the frequency, and $c$ the speed of light;

2. The Fabry–Pérot condition^30^:

2 $${\varphi }_{{\Gamma }_{sup}}+{\varphi }_{{\Gamma }_{refl}}-2k{h}_{FP}=-2N\pi$$

where ${\varphi }_{{\Gamma }_{sup}}$ is the phase of the reflection coefficient associated to the superstrate;

3. The topology constraint:

3 $${h}_{FP}>{h}_{refl}$$

The wavenumber $k$ can considerably vary in a multiband operation while ${h}_{refl}$ and ${h}_{FP}$ are fixed. Hence, the metasurfaces should provide phases ${\varphi }_{{\Gamma }_{sup}}$ and ${\varphi }_{{\Gamma }_{refl}}$ that meet conditions I–III given ${h}_{refl}$, ${h}_{FP}$, and the center frequency ${f}_{c}$ of each band. This task is as easier as ${\varphi }_{{\Gamma }_{sup}}$ and ${\varphi }_{{\Gamma }_{refl}}$ are adjustable in each band of operation. However, the functionalities that one may wish to extract from the metasurfaces can constrain ${\varphi }_{{\Gamma }_{sup}}$ and ${\varphi }_{{\Gamma }_{refl}}$.

### Topology for validation

**Figure 2:**
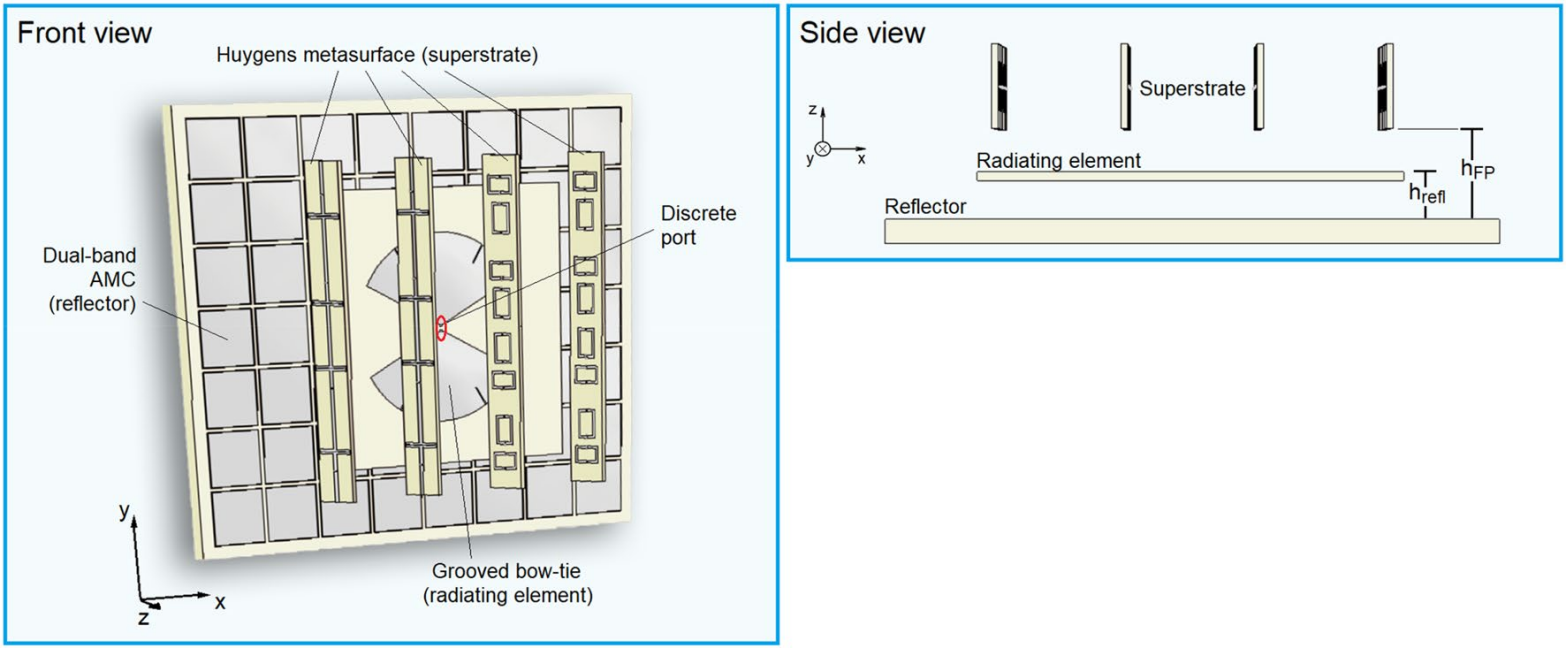
Topology with floating structures and discrete port.

We propose a topology that is not intended to be a Fabry–Pérot cavity but uses a grooved bow-tie antenna^31^ as the radiating element, surrounded by a dual-band AMC^32^ as reflector, and a Huygens metasurface^33^ as superstrate. In order to simplify the problem and highlight the key point of the undesired Fabry–Pérot resonances, we first consider a topology with discrete port and floating structures (Fig. 2). The grooved bow-tie antenna, detailed in Supplementary Fig. 1, has a wideband behavior with stable radiation patterns with maxima at each broadside direction in the B1–B4 bands. The dual-band AMC (Supplementary Note 1) has $7\times 8$ cells. Near resonance frequencies, its reflection coefficient phase ${\varphi }_{{\Gamma }_{refl}}\left(f\right)$ can be easily chosen during the design. Far from resonances such phase is not so settable and asymptotically goes to ${\varphi }_{{\Gamma }_{refl}}\left(f\right)=\pm 180^\circ$. As such, a multiband reflection scheme can be conceived to provide the phases required by conditions I–III and make the antenna directive in all the bands of interest B1–B4. The Huygens metasurface (Supplementary Note 2) uses varactors to control the transmission coefficient phase ${\varphi }_{{\tau }_{sup}}$ in the B2 band without disturbing such phase in the other bands. This capability could be used to shape the radiation pattern and increase its directivity at 5G frequencies with the correct choice of the phases of each unit cell^13–18^. However, in this work, the antenna exploits a phase-gradient mechanism to steer the antenna beam at 5G frequencies, while the beam remains fixed at other frequencies. The difference in relation to previous phase-gradient works^10–14^ is that the unintended Fabry–Pérot cavity and the proximity of the radiation source and the superstrate require an optimization of the varactor values to achieve a specific steering angle. Since we do not intend to create a Fabry–Pérot cavity, such metasurface does not need to cover the whole device. Therefore, we use the minimum number of cells ($4\times 4$) that allows for the beam-steering functionality. Adjusting the Huygens metasurface reflection coefficient phase ${\varphi }_{{\Gamma }_{sup}}$ is not easy and impact its transmission coefficient ${\varphi }_{{\tau }_{sup}}$.

### Taming Fabry–Pérot resonances in multiple bands

The methodology consists of the following steps:

- define a range of possible values for the spacing ${h}_{refl}\in \left[{h}_{ref{l}_{1}},{h}_{ref{l}_{2}}\right]$ between reflector and radiating element according to the frequency band where the reflection phase ${\varphi }_{{\Gamma }_{refl}}$ is the hardest to adjust; from condition I, with $N=-1$:
  4 $$\frac{c}{{f}_{l}}\left[\frac{{\varphi }_{{\Gamma }_{refl}}\left({f}_{l}\right)}{4\pi }+\frac{1}{3}\right]<{h}_{refl}<\frac{c}{{f}_{h}}\left[\frac{{\varphi }_{{\Gamma }_{refl}}\left({f}_{h}\right)}{4\pi }+\frac{2}{3}\right]$$
  where ${f}_{l}$ and ${f}_{h}$ are the lowest and highest frequencies of the specific band.
- For each other band, define the range of reflection phases ${\varphi }_{{\Gamma }_{refl}}\in \left[{\varphi }_{{\Gamma }_{ref{l}_{1}}},{\varphi }_{{\Gamma }_{ref{l}_{2}}}\right]$ constrained by condition I, considering that $\left[{h}_{ref{l}_{1}},{h}_{ref{l}_{2}}\right]$ was already defined:
  5 $$-\frac{2\pi }{3}+\frac{4\pi {f}_{c}}{c}{h}_{ref{l}_{1}}+2N\pi <{\varphi }_{{\Gamma }_{refl}}<+\frac{2\pi }{3}+\frac{4\pi {f}_{c}}{c}{h}_{ref{l}_{2}}+2N\pi$$
- According to the range $\left[{\varphi }_{{\Gamma }_{ref{l}_{1}}},{\varphi }_{{\Gamma }_{ref{l}_{2}}}\right]$ for each band, define a range of possible values for the spacing ${h}_{FP}\in \left[{h}_{F{P}_{1}},{h}_{F{P}_{2}}\right]$ between reflector and superstrate; from condition II, we have:
  6 $$\frac{c}{4\pi f}\left({\varphi }_{{\Gamma }_{sup}}+{\varphi }_{{\Gamma }_{ref{l}_{1}}}+2\pi N\right)<{h}_{FP}<\frac{c}{4\pi f}\left({\varphi }_{{\Gamma }_{sup}}+{\varphi }_{{\Gamma }_{ref{l}_{2}}}+2\pi N\right)$$
  Due to condition III, disregard values ${h}_{FP}\le {h}_{ref{l}_{1}}$. Then, pick a final range $\left[{h}_{F{P}_{1}},{h}_{F{P}_{2}}\right]$ which is the intersection of the ranges for each band.
- for each band where the reflection coefficient phase ${\varphi }_{{\Gamma }_{refl}}$ is easy to adjust, define a new range $\left[{\varphi }_{{\Gamma }_{ref{l}_{1}}},{\varphi }_{{\Gamma }_{ref{l}_{2}}}\right]$ that respects condition II given the final range $\left[ {h_{{FP_{1} }} ,h_{{FP_{2} }} } \right]$:
  7 $$\frac{{4\pi f}}{c}h_{{FP_{1} }} - \varphi _{{\Gamma _{{\sup }} }} - 2\pi N < \varphi _{{\Gamma _{{refl}} }} < \frac{{4\pi f}}{c}h_{{FP_{2} }} - \varphi _{{\Gamma _{{\sup }} }} - 2\pi N$$
  After that, we evaluate a new range for the spacing ${h}_{refl}$ considering condition I and the center frequency ${f}_{c}$ of each band not addressed in step (a). Then, we take a final value for ${h}_{refl}$ in the intersection of all these ranges.

Considering the features of the dual-band AMC and Huygens metasurface of Supplementary Notes 1 and 2, and then following steps (a)–(d), we obtained the parameters of Table 1. This process is detailed at Supplementary Note 3. These parameters are a starting point for the complete design, which should be optimized with full-wave simulations that consider the actual incidence of waves over the finite AMC and superstrate.

**Table 1:** Parameters defined by the methodology.

Symbol	Description	Value
${h}_{FP}$	Distance between the superstrate and the reflector	$15.1\; {\text{mm}}$
$h_{refl}$	Distance between the reflector and the radiating element	$12.9 \;{\text{mm}}$
$\varphi_{{\Gamma_{refl} }}$ (B1)	Reflection coefficient phase of the reflector in the B1 center frequency ($2.55 \;{\text{GHz}}$)	$+ 24.2^\circ$
$\varphi_{{\Gamma_{refl} }}$ (B2)	Reflection coefficient phase of the reflector in the B2 center frequency ($3.60 \;{\text{GHz}}$)	$- 128.5^\circ$
$\varphi_{{\Gamma_{refl} }}$ (B3 + B4)	Reflection coefficient phase of the reflector in the B3 + B4	$- 160.0^\circ$

### Checking the relevance of Fabry–Pérot resonances

**Figure 3:**
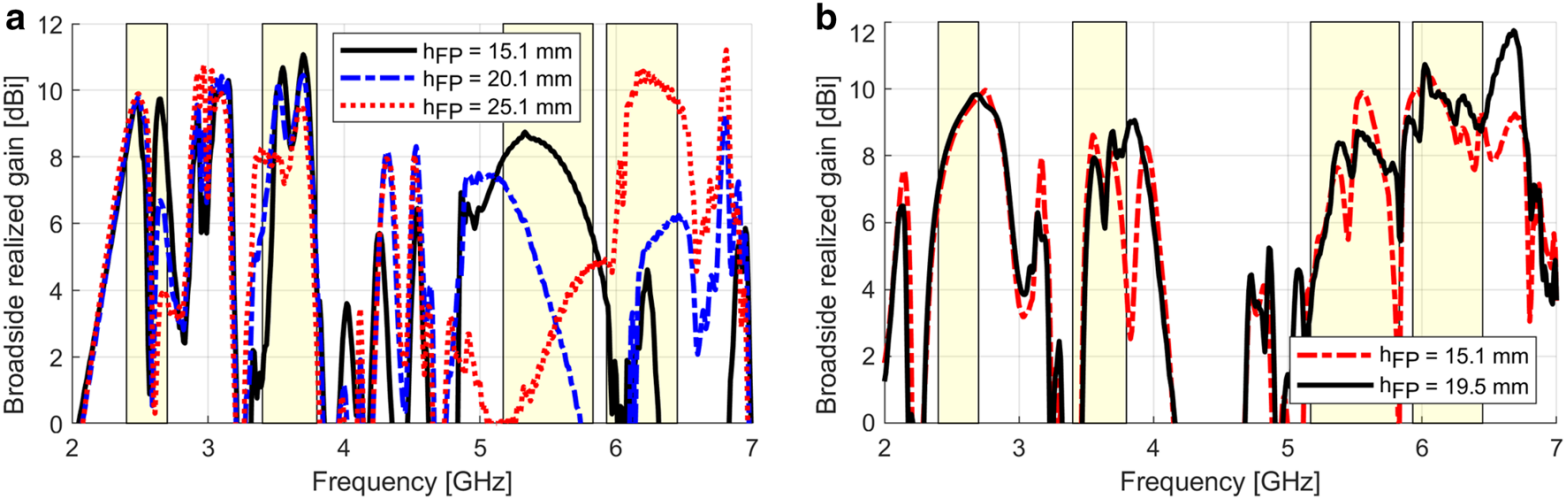
Simulated broadside realized gain when the ${h}_{FP}$ is varied; the bands B1–B4 are highlighted. (**a**) Topology of Fig. 2. (**b**) Topology of Fig. 4, after optimization. The B1–B4 bands are highlighted.

To highlight the relevance of Fabry–Pérot resonances, we first simulated the topology of Fig. 2, which does not present a feeding system or mechanical supports. The AMC unit cell was optimized as in Supplementary Fig. 4 to approach the phases ${\varphi }_{{\Gamma }_{refl}}$ of Table 1. The grooved bow-tie and the Huygens unit cell whose parameters are listed in Supplementary Tables 1 and 3 were also used. Similar to this recent work^34^, the varactor capacitances $C$ of each column of the Huygens metasurface were adjusted to perform a near-field phase compensation. The relevance of Fabry–Pérot resonances becomes clear when changes are seen in the broadside gain due to variations of the spacing ${h}_{FP}$ because the interference patterns between bouncing waves transmitted through the superstrate depend on ${h}_{FP}$. The distance ${h}_{refl}$ was $12.9\,\mathrm{mm}$ and ${h}_{FP}$ was varied from $15.1$ to $25.1\,\mathrm{mm}$. Figure 3a shows a clear influence of ${h}_{FP}$ on the results in all the B1–B4 bands. Increasing ${h}_{FP}$ makes the broadside gain decrease in the second half of the B1, in most of the B2 and in the first half of the B3 band. Conversely, the gain in the first half of the B1 and in most of the B4 band increases. Thus, even if the superstrate is not covering the whole of the reflector and the superstrate reflection coefficient magnitude is low for most of the bands (see Supplementary Fig. 7a), Fabry–Pérot resonances significantly impact performance. The result for ${h}_{FP}=15.1\,\mathrm{mm}$ is satisfactory for a first simulation since good levels of gain were indeed reached in each of the B1, B2 and joint B3 + B4 bands (even if not in the whole bands), making evident the benefits of the proposed methodology. However, drops of gain in the second half of the B1 and joint B3 + B4 bands are seen, mainly because the incidence of waves over the AMC and the superstrate in the complete design is different from that in the simulation of the standalone AMC and Huygens unit cells. Hence, an optimization of the topology of Fig. 2 was performed with full-wave simulations as detailed in Supplementary Note 4.

**Figure 4:**
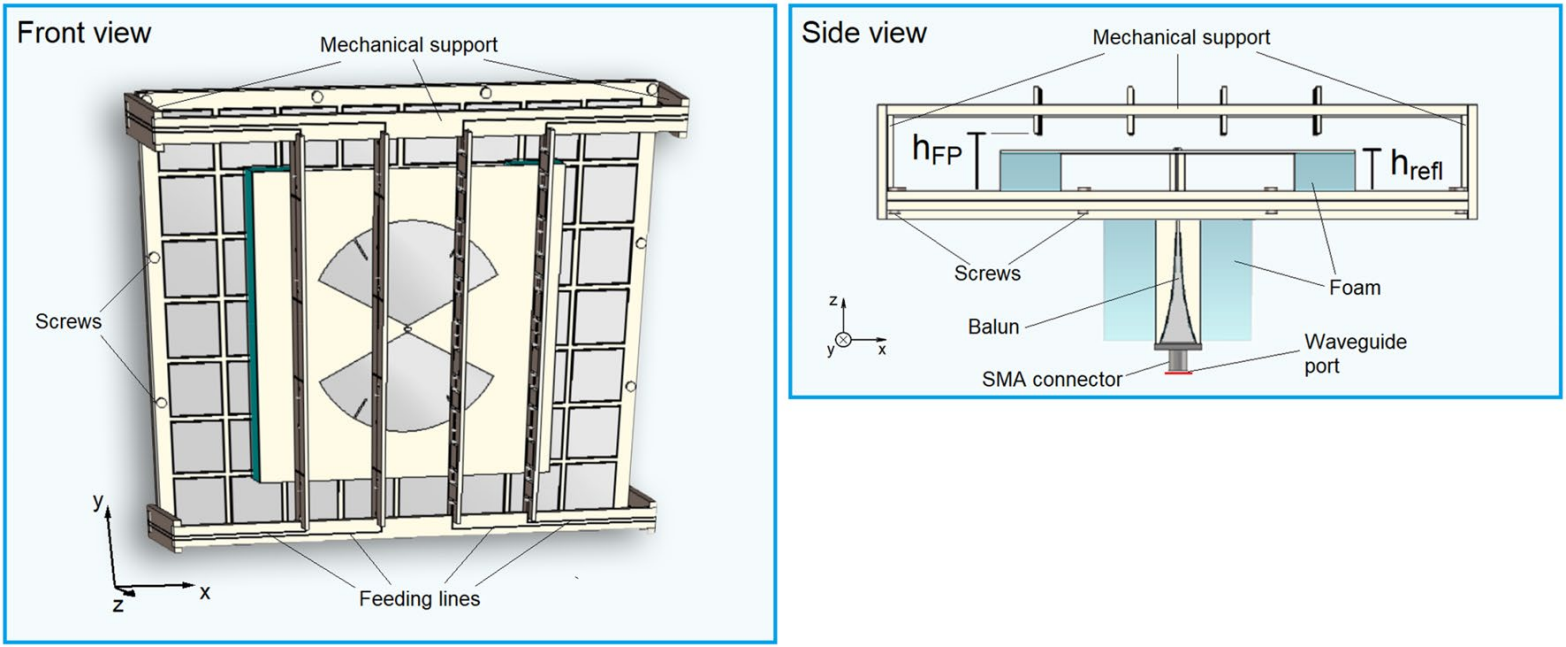
Complete design.

### Fabrication process

Feeding lines were inserted in the Huygens metasurface to bias the varactors to control the transmission coefficient phase ${\varphi }_{{\tau }_{sup}}$. To allow the measurements in an anechoic chamber with a $50$-$\Omega$ SMA connector, an exponential taper balun was introduced, as well as mechanical supports. Foam bricks were placed around the balun and between the bow-tie antenna and the AMC for mechanical stability. Teflon screws were used to fix two sheets of dielectric laminates used in the AMC. The insertion of these structures is detailed in Supplementary Note 5 and Fig. 4. Figure 3b shows the simulated broadside realized gain for this final design. For ${h}_{FP}=15.1\,\mathrm{mm}$, peak values of $10.0$, $8.5$, $10.0$, and $10.3\,\mathrm{dBi}$ are respectively seen in the B1–B4 bands.

As a last step before fabrication, we optimized the spacing $h_{FP}$ between the superstrate and the AMC in an attempt to improve the gain stability in the B3 and B4 bands. Figure 3b also shows the simulated results for an optimal $h_{FP} = 19.5\; {\text{mm}}$. The B1 and B2 bands present gain values comparable to the case $h_{FP} = 15.1 \;{\text{mm}}$. In the B3 and B4 bands, a higher stability is indeed seen. In the beginning of the B3 band, the gain is in a steep climb, crossing $5.17 \;{\text{GHz}}$ with $3.4\; {\text{dBi}}$ and keeping around $8.0 \;{\text{dBi}}$ from $5.30\; {\text{GHz}}$ until the band frequency limit ($5.83 \;{\text{GHz}}$), where a drop occurs. In the B4, levels around $9.5 \;{\text{dBi}}$ are seen. Considering thicknesses of $4.9 \;{\text{mm}}$ for the AMC, $12.25 \;{\text{mm}}$ for the superstrate, and $h_{FP} = 19.5 \;{\text{mm}}$, the final device is $0.29 \lambda_{l}$ thick, where $\lambda_{l}$ is the wavelength at $2.4 \;{\text{GHz}}$. The balun is not considered in this calculation because, in a real application, the feeding of the antenna would occur through a balun integrated in a board parallel to the antenna ground plane. Last, the aperture size is $136.1 \times 154.4 \;{\text{mm}}^{2}$, that is, $1.1 \times 1.2 \lambda_{l}^{2}$.

**Figure 5:**
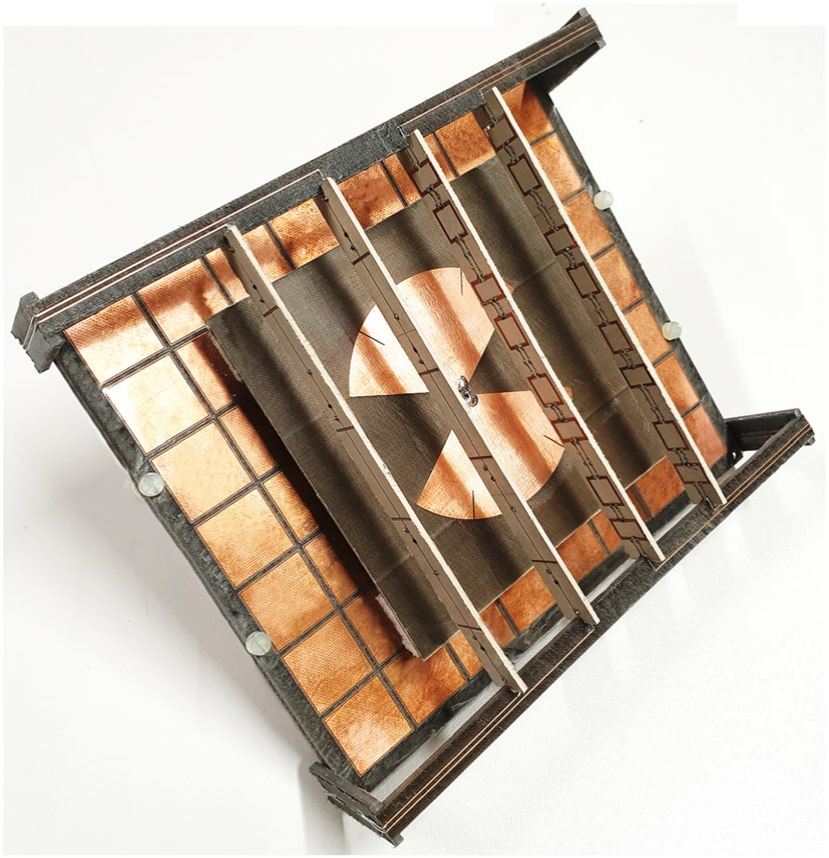
Fabricated device.

The assembling of the prototype was done at Télécom Paris, including a difficult stage of manually welding 80 varactors and 184 resistors in the Huygens elements with the aid of a microscope. The final prototype, shown in Fig. 5, was simulated and measured in five different beam-steering states ($- 2$, $- 1$, $0$, $+ 1$, $+ 2$) set through different bias voltages on the superstrate varactors. More details on the assembling and biasing are given in Supplementary Note 6.

### Measurements

**Figure 6:**
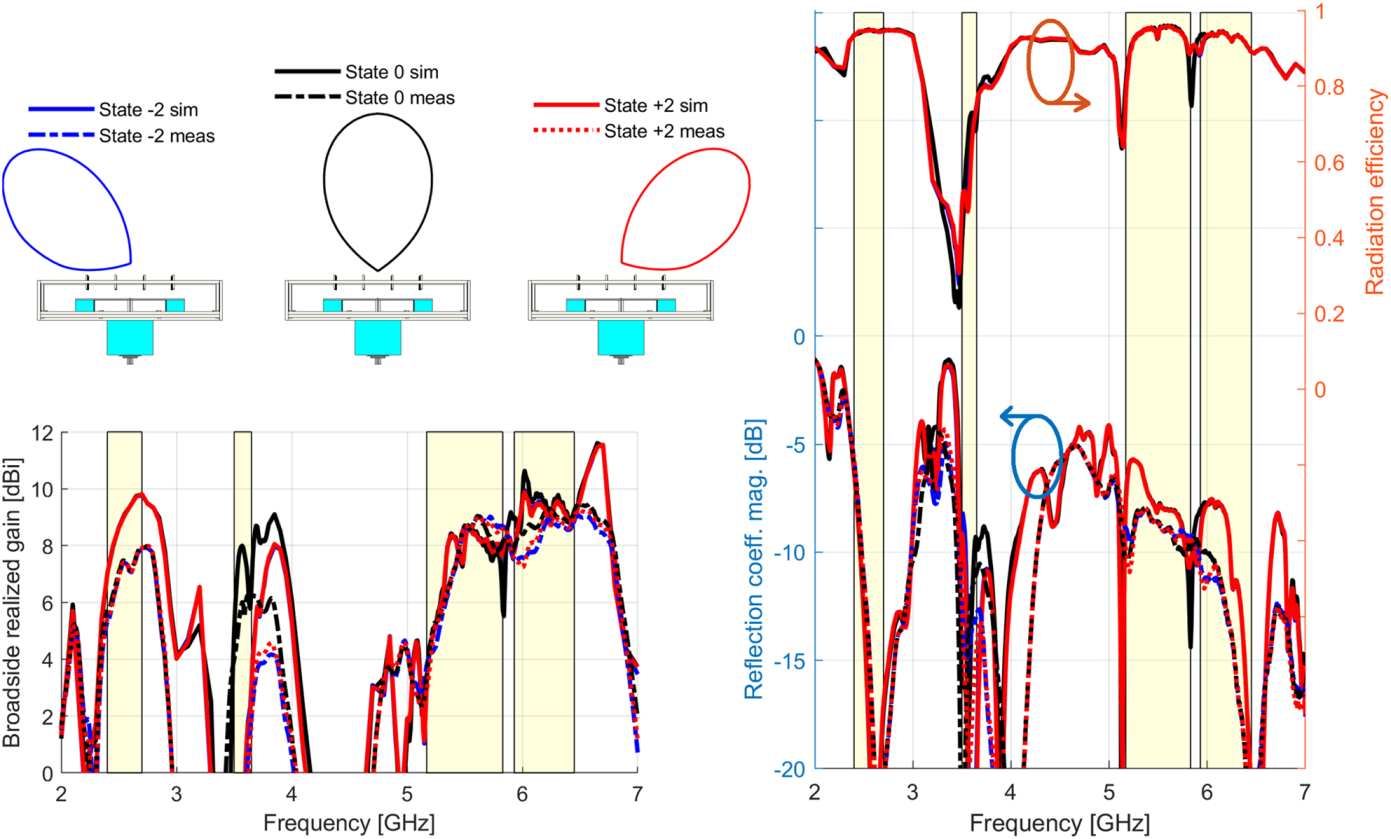
Broadside realized gain, reflection coefficient magnitude |Γ|, and simulated radiation efficiency for the states − 2, 0, and + 2.

The broadside realized gain is shown in Fig. 6. Inside the B2 band, the beam-steering is optimal in the range 3.50–3.65 GHz, which is highlighted with the B1, B3, and B4 bands. For the sake of clarity, only the extreme and center beam-steering states ($- 2$, $0$, and $+ 2$) are shown now. For the B1 band, the peak gain was simulated as $9.8\; {\text{dBi}}$ and measured as $7.9 \;{\text{dBi}}$ for the states $- 2$, $0$, and $+ 2$. In the range 3.50–3.65 GHz, the peak gain of the state $0$ was simulated as $8.0 \;{\text{dBi}}$ and measured as $6.0 \;{\text{dBi}}$ (note that the other states radiate mostly at other directions). For the B3 and B4 bands, peak gains are respectively simulated as $8.7$ and $10.6\; {\text{dBi}}$ and measured as $9.0$ and $9.2 \;{\text{dBi}}$. These results are good considering the in-house fabrication process with the manual welding of the components.

For reference, we also show in Fig. 6 the reflection coefficient magnitude $\left| \Gamma \right|$ and the simulated radiation efficiency, which were both already intrinsically computed in the realized gain. For the reflection coefficient magnitude $\left| \Gamma \right|$, simulation and measurement agree particularly well in the B1 band. For the states $- 2$, $0$, and $+ 2$, $\left| \Gamma \right| = - 5.7\,{\text{dB}}$ at $2.40 \,{\text{GHz}}$ and drastically drops for $- 20.0 \,{\text{dB}}$ at $2.60 \,{\text{GHz}}$. The agreement is also good in the range $3.50$–$3.65\, {\text{GHz}}$ in the B2 band, where the simulated and measured reflection coefficient magnitudes are respectively below $- 8.2$ and $- 10.9 \,{\text{dB}}$ for the three states. In the B3 band, the measurements ($\le - 8.0 \,{\text{dB}}$) present slightly better values than the simulations ($\le - 5.7 \,{\text{dB}}$). The same happens in the B4 band (measured as $\le - 9.8 \,{\text{dB}}$ versus simulated as $\le - 7.5\, {\text{dB}}$). The fact that the reflection coefficient magnitude $\left| \Gamma \right|$ keeps stable with the changing between steering states indicates that a simple matching network can be engineered to enhance it if some application requires so. However, the current work is focused on achieving a directive, multiband behavior in the presence of undesired Fabry–Pérot resonances. Hence, to avoid losing focus on the interferences of waves due to Fabry–Pérot resonances, we considered these results satisfactory to validate our findings and the proposed methodology (also because they were already taken into account in the realized gain, which is good considering the aperture size of $1.1 \times 1.2 \lambda_{l}^{2}$). Further, the simulated radiation efficiency shows values above $0.9$ for all the B1, B3, and B4 bands. In the B2 band, the radiation efficiency keeps around $0.6$ for the three states due to losses in the varactors. Again, this result is already taken into accounted in the realized gain and is considered satisfactory for the purposes of this work.

**Figure 7:**
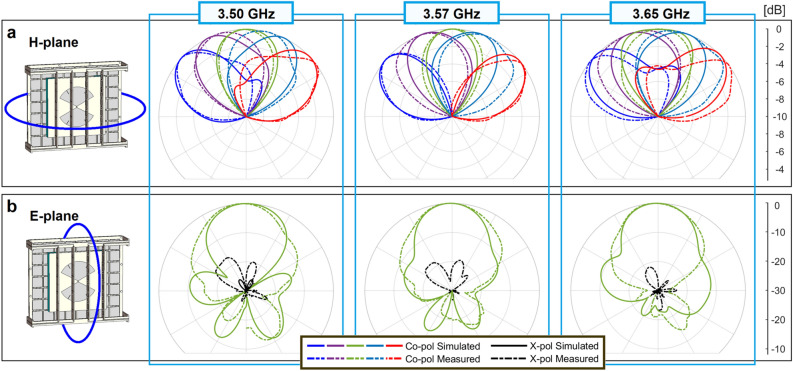
Normalized radiation patterns in the range 3.50–3.65 GHz in the B2 (**a**) all states in the H-plane. (**b**) State 0 in the E-plane.

To highlight the beam-steering, Fig. 7a shows the radiation patterns in the H-plane from $3.50$ to $3.65\, {\text{GHz}}$ with a scale of $10 \,{\text{dB}}$. A very good agreement is seen between simulations and measurements, mainly for the states $- 2$, $0$, and $+ 2$. The capacitance $C$ in the simulations and the bias voltages in the measurements were harder to match in the states $- 1$, and $+ 1$ because they were intermediate values in their possible ranges. Figure 7b shows the radiation patterns for the state $0$ in the E-plane with a scale of $30\, {\text{dB}}$. For all the states in the H-plane and state $0$ in the E-plane, from $3.50$ to $3.65\, {\text{GHz}}$, the cross-polarization level keeps better than $- 18.7$, $- 23.3$, and $- 23.0 \,{\text{dB}}$ in the simulations versus $- 13.5$, $- 13.6$, and $- 16.2 \,{\text{dB}}$ in the measurements. The front-to-back ratio was respectively better than $13.2$, $19.2$, and $17.4 \,{\text{dB}}$ in the simulations and $16.4$, $15.7$, and $16.2 \,{\text{dB}}$ in the measurements.

**Figure 8:**
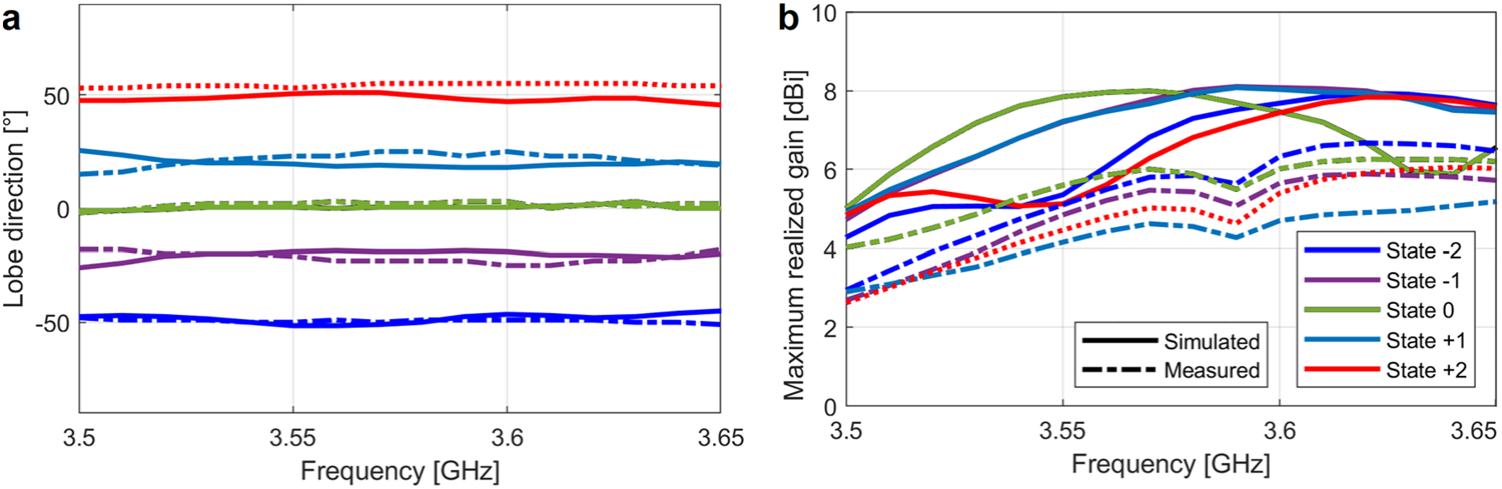
Beam-steering results in the range 3.50–3.65 GHz. (**a**) Lobe direction. (**b**) Maximum realized gain.

**Figure 9:**
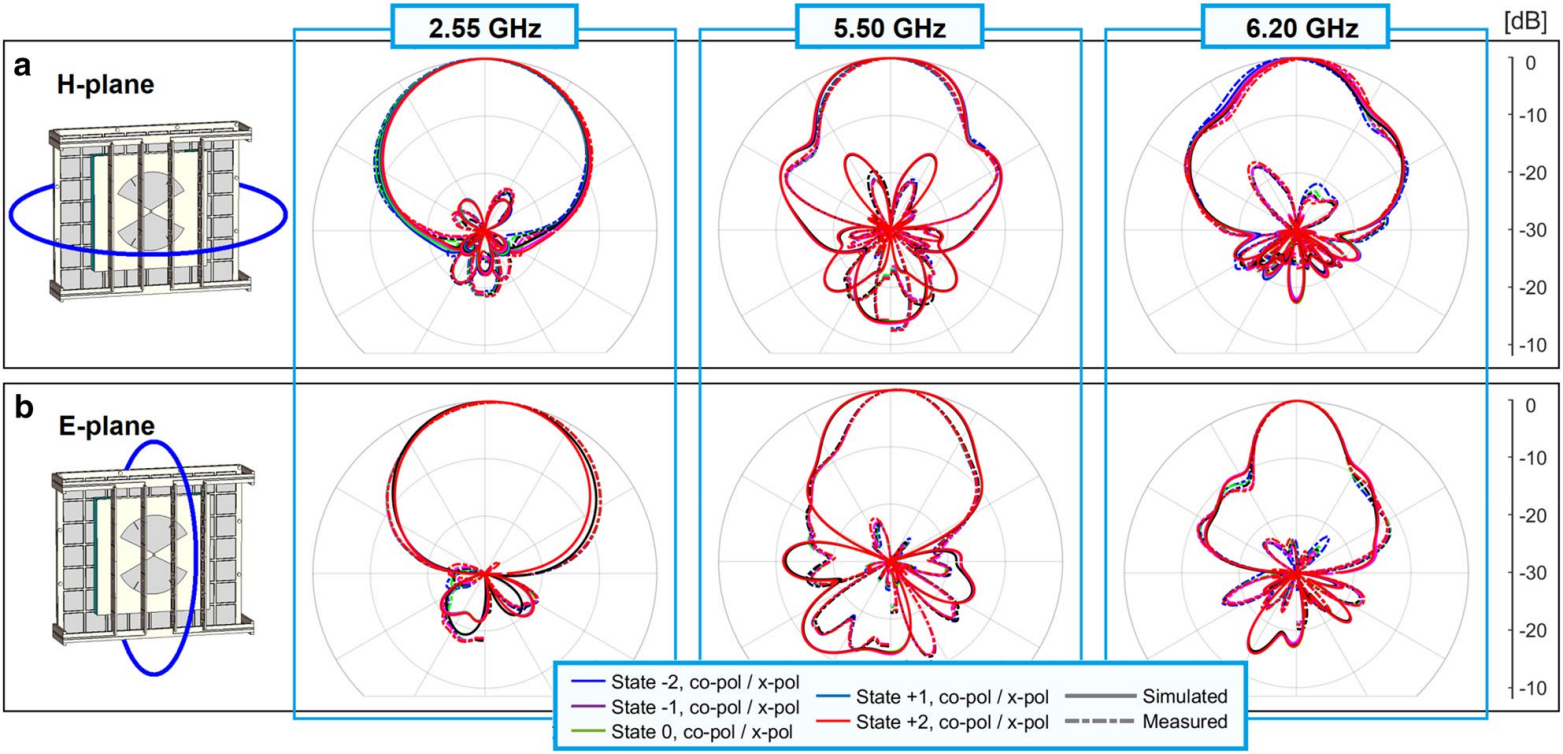
Normalized radiation patterns for the bands B1, B3, and B4.

Figure 8a shows the lobe direction in the range $3.50$–$3.65\, {\text{GHz}}$ when the beam is steered in the H-plane. Again, simulations and measurements agree very well, considering the imprecisions in the prototype due to the in-house fabrication process. Figure 8b shows the maximum realized gain in the same conditions. Simulations presented peak values around $8 {\text{dBi}}$ for all the states while in the measurements the gain reaches around $6 {\text{dBi}}$ for the states $- 2$, $- 1$, $0$, and $+ 2$, and $5 {\text{dBi}}$ for the state $+ 1$. All the states keep in the gain range $3.0$–$6.0\,{\text{dBi}}$ from $3.50$ to $3.65\,{\text{GHz}}$, except the state $- 2$, that surpass the $6.0$-${\text{dBi}}$ level from $3.60\,{\text{GHz}}$. We consider therefore the instantaneous 3-dB gain bandwidth as $4.2\%$. Figure 9 shows the radiation patterns in the B1, B3 and B4 bands. Simulations and measurements agree well in both H- and E-planes. Two main points should be noted in these patterns:

- the patterns present shapes typical of directive antennas, without deformations associated to untamed Fabry–Pérot resonances;
- the shapes of the radiation patterns do not change when we migrate from one state to another.

## Discussion

The key point (1) above validates our methodology, which, followed by the optimization of the whole device, indeed worked well for the control of undesired Fabry–Pérot resonances in multiple bands. The key point (2) indicates that the beam-steering could be performed in the range $3.50$*–*$3.65\,{\text{GHz}}$ for the 5G standard without disturbing the operation in the other bands. The above results show that, by using the proposed methodology, we succeeded in exploiting the functionalities of two metasurfaces in multiple bands despite the presence of Fabry–Pérot resonances. These functionalities are, from the side of the Huygens metasurface, the beam-steering in the 5G without disturbing the other bands, and, from the side of the dual-band AMC, a multiband reflection scheme and a thickness of only $0.29 \lambda_{l}$, ($\lambda_{l}$ is the wavelength at $2.4\,{\text{GHz}}$). We do not see any limitation on applying the presented concepts for other combinations of functionalities. As long as conditions I–III are respected, a stable radiation pattern with a maximum gain in the broadside direction will be obtained in each of the operating bands. We also believe that these concepts may be applied to other areas, such as Acoustics, and Elastodynamics, which may lead to future studies on compact, multiband, beam-steerable microphones or sonar transducers using two parallel metasurfaces, among others.

## Methods

### Simulation methodology

The transient solver of the CST Studio Suite was used in all simulations. In the simulation of the unit cells of the AMC and the Huygens metasurface, perfect electric conductor and perfect magnetic conductor boundary conditions are used together with waveguide ports to emulate an infinite array a transverse electromagnetic wave impinges upon with normal incidence. In the simulation of the model of Fig. 2, a discrete port with reference impedance $Z_{ref} = 151 \;\Omega$ is used in the bow-tie input terminals. In the simulation of the model of Fig. 4, a waveguide port is set over the balun SMA connector.

### Measurement methodology

The measurements of the radiation patterns and the reflection coefficient of the fabricated prototype took place in the anechoic chamber of Télécom Paris, using an Agilent N5230C PNA-L network analyzer, which operates from $10\,{\text{MHz}}$ to $50\,{\text{GHz}}$. The bias voltages for the four columns of the Huygens metasurface were provided by two Hewlett Packard dual-power supplies model E3620A. The power supplies were placed outside the chamber, together with the network analyzer, and the voltage were applied via $0.2$-${\text{mm}}$ diameter copper wires.

## Supplementary Information

Supplementary Information.

## Data Availability

The data that support the findings of this study are available from the corresponding author on request.
