# Supplementary material for: Taming Fabry–Pérot resonances in a dual-metasurface multiband antenna with beam steering in one of the bands
Source: Sci Rep. 2023 Jun 19;13:9871. doi: 10.1038/s41598-023-36828-4 (PMC10279765; doi:10.1038/s41598-023-36828-4)
Supplement: Supplementary file 1 — Supplementary Information. [file 41598_2023_36828_MOESM1_ESM.docx]

Supplementary Information for
Taming Fabry-Pérot Resonances in a dual-metasurface
multiband antenna with beam steering in one of the bands

Rafael Gonçalves Licursi de Mello ^1*^, Anne Claire Lepage ^1^, Xavier Begaud ^1^

^1^ LTCI, Télécom Paris, Institut Polytechnique de Paris, Palaiseau, France.

^*^Corresponding author: [rafael.licursi@ieee.org](mailto:rafael.licursi@ieee.org)

Supplementary Fig. 1 illustrates a grooved bow-tie antenna^31^ that was adjusted and optimized for impedance matching purposes.

**
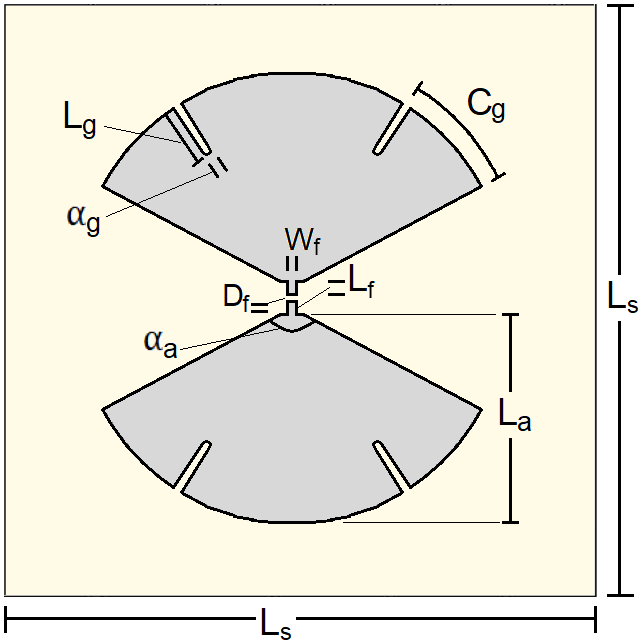
**

**Supplementary Fig. 1.** Grooved bow-tie used in the current work.

Without a reflector or superstrate, the optimal reference impedance to match the grooved bow-tie antenna^31^ is $Z_{ref}=185 Ω$. The introduction of a reflector and a superstrate, however, changes the input impedance of the antenna, as expected. Supplementary Table 1 summarizes the parameters of the employed antenna.

**Supplementary Table 1.** Parameters of the grooved bow-tie used in the current work

| **Symbol** | **Description** | **New value** |
| --- | --- | --- |
| $\varepsilon_{r}$ | substrate relative permittivity | $2.2$ |
| $\tan\delta$ | substrate loss tangent | $0.0009$ |
| $L_{s}$ | substrate length | $94.5 mm$ |
| $t_{s}$ | substrate thickness | $0.76 mm$ |
| $L_{a}$ | bow length | $31.5 mm$ |
| $\alpha_{a}$ | bow flare angle | $124.4^{\circ}$ |
| $L_{f}$ | feeding strip length | $0.80 mm$ |
| $W_{f}$ | feeding strip width | $0.60 mm$ |
| $D_{f}$ | gap between feeding strips | $0.76 mm$ |
| $L_{g}$ | groove length | $7.0 mm$ |
| $\alpha_{g}$ | groove angular width | $0.69^{\circ}$ |
| $C_{g}$ | groove angular position | $30.0^{\circ}$ |

**Supplementary Note 1. The dual-band AMC**

The double-square unit cell over a ground plane was used in this work because among many other shapes it was the most appropriate to provide the phases required by the methodology to tame Fabry-Pérot resonances in multiple bands and because its simple geometry without curves makes easier to simulate the whole structure. A detailed study on the double-square AMC, including results for finite arrays of $6\times6$, $8\times8$, and $10\times10$ cells, was recently presented^32^. Supplementary Fig. 2 shows such unit cell geometry as well as its parameters.


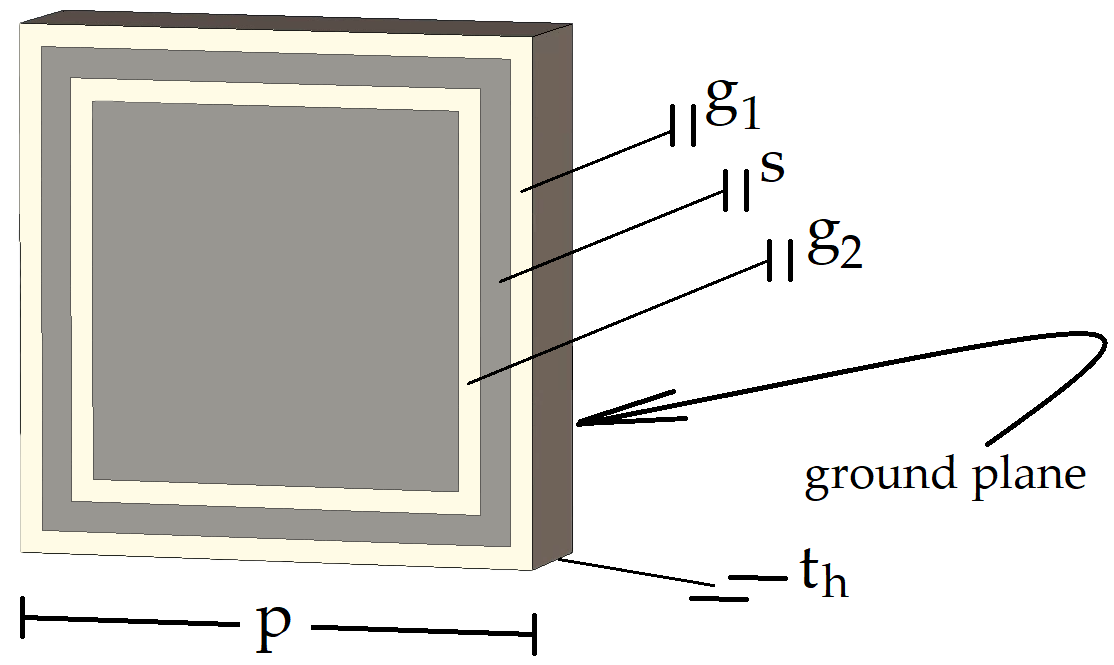


**Supplementary Fig. 2.** AMC unit cell and its parameters (reproduced with authorization^32^).

In that study^32^, the role of the AMC was not the same as in the current work. Such role was to enhance the broadside gain of the antenna by respecting only the condition^29^ $-120^{\circ}<\varphi_{t}<+120^{\circ}$ in each frequency band of operation, where $\varphi_{t}$ is the phase difference between direct and electric fields in the plane of the antenna. Nevertheless, that study shows how the unit cell can be optimized to provide specific reflection phases. Moreover, a study on the currents of the $8\times8$-cell AMC justifies the reason the final number of cells is $7\times8$ cells.

In the current work, conditions I–III should be respected, and the AMC is a key element because it can provide arbitrary phases close to the ones calculated with the proposed methodology adjusting thereby the Fabry-Pérot resonances in each of the B1–B4 bands. Supplementary Fig. 3 illustrates the role of the AMC in the current work.

**
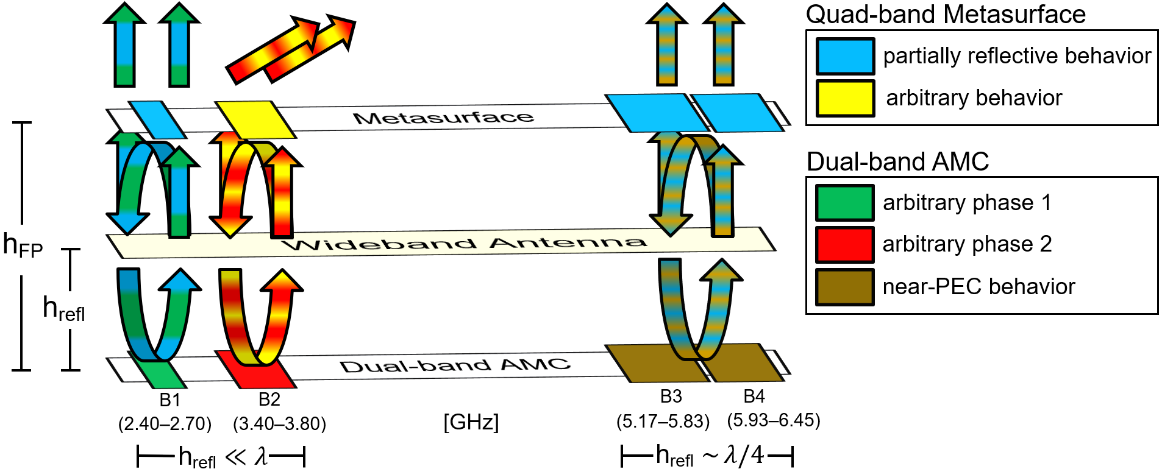
**

**Supplementary Fig. 3.** The AMC role in this work

: the metasurface superstrate is not perfectly transparent; the AMC provides arbitrary phases to handle the resulting Fabry-Pérot resonances in each of the B1–B4 bands.

In main-text Table 1, ideal values for the AMC reflection phase are defined as $\varphi_{\Gamma_{refl}}=+24.2^{\circ}$ in the B1 band, $\varphi_{\Gamma_{refl}}=-128.5^{\circ}$ in the B2 band, and $\varphi_{\Gamma_{refl}}=-160.0^{\circ}$ in the B3+B4 joint band. Supplementary Table 2 shows the evolution of parameters of the double-square unit cell to obtain such phases.

**Supplementary Table 2.** Parameters of unit cell intended to match the phases of Table 1

| **Symbol** | **Description** | **Single-role AMC**^32^ | **Fabry-Pérot-adjusting AMC** |
| --- | --- | --- | --- |
| $\varepsilon_{r}$ | substrate relative permittivity | $2.34$ | $2.34$ |
| $g_{1}$ | outer gap | $0.58 mm$ | $0.65 mm$ |
| $g_{2}$ | inner gap | $0.30 mm$ | $0.10 mm$ |
| $p$ | periodicity | $17.5 mm$ | $20.5 mm$ |
| $s$ | strip between gaps | $0.25 mm$ | $0.10 mm$ |
| $\tan\delta$ | substrate loss tangent | $0.0013$ | $0.0013$ |
| $t_{h}$ | substrate thickness | $4.9 mm$ | $4.9 mm$ |

Supplementary Fig. 4 shows the evolution of the reflection coefficient phase $\varphi_{\Gamma_{refl}}$ of the unit cell from the single-role AMC^32^ to the AMC used in this work. The simulation was performed with the transient solver of CST Microwave Studio with proper PEC and PMC boundary conditions to emulate an infinite array. In this configuration, a TEM wave illuminates the surface with normal incidence.


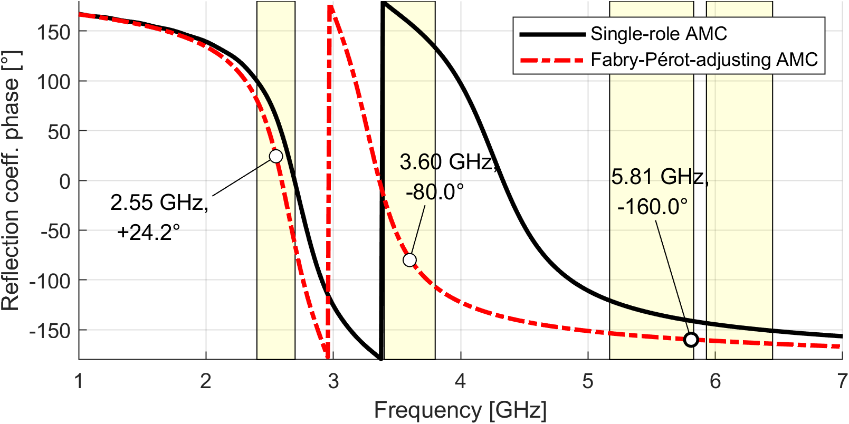


**Supplementary Fig. 4.** Reflection coefficient phase $\varphi_{\Gamma_{refl}}$ of the double-square element

intended to match the phases of Table 1.

In Supplementary Fig. 4, it is clear that the Fabry-Pérot-adjusting unit cell can provide the exact desired reflection coefficient phases $\varphi_{\Gamma_{refl}}$ for the central frequencies of the B1 and joint B3+B4 bands. However, for the B2 band, $\varphi_{\Gamma_{refl}}=-80.0^{\circ}$ instead of $-128.5^{\circ}$. The parameters summarized in Table 1 are a starting point for the complete design, which should be optimized through full wave simulations which take into account the actual incidence of waves over the AMC and the superstrate in the final design. Therefore, such phases were considered acceptable at this stage.

**Supplementary Note 2. The Huygens metasurface**

A reconfigurable two-layer Huygens unit cell was recently realized^33^ with the insertion of controllable active elements, specifically varactors. The following reasons made us choose such metasurface as a superstrate for our topology:

1. the large number of parameters we can act over in its unit cell allows us to tailor the performance of the metasurface in multiple bands;
2. Huygens metasurface allow a good transmission coefficient magnitude and control of phase;
3. the fact that Huygens metasurface are well-known and extensively reported in the literature allows us to keep focused on the methodology to tame Fabry-Pérot resonances in multiple bands, ultimately facilitating its use;
4. The above reasons were necessary and sufficient to illustrate the presented concepts and the proposed methodology;
5. Besides, such metasurface requires a low number of unit cells in our design, making the control of the beam-steering easy.

One of the layers of this unit cell is composed of two symmetrical split rings, each comprising a varactor of capacitance $C$. The other is made of a cut wire with a capacitor-type structure and three varactors also of value $C$. In our simulations, a wave with electric field $\mathbf{E}^{\boldsymbol{i}}=\mathbf{â}_{\boldsymbol{y}}E_{0}e^{-jkz}$ is set to impinge upon such unit cell, illustrated in Supplementary Fig. 5.

**
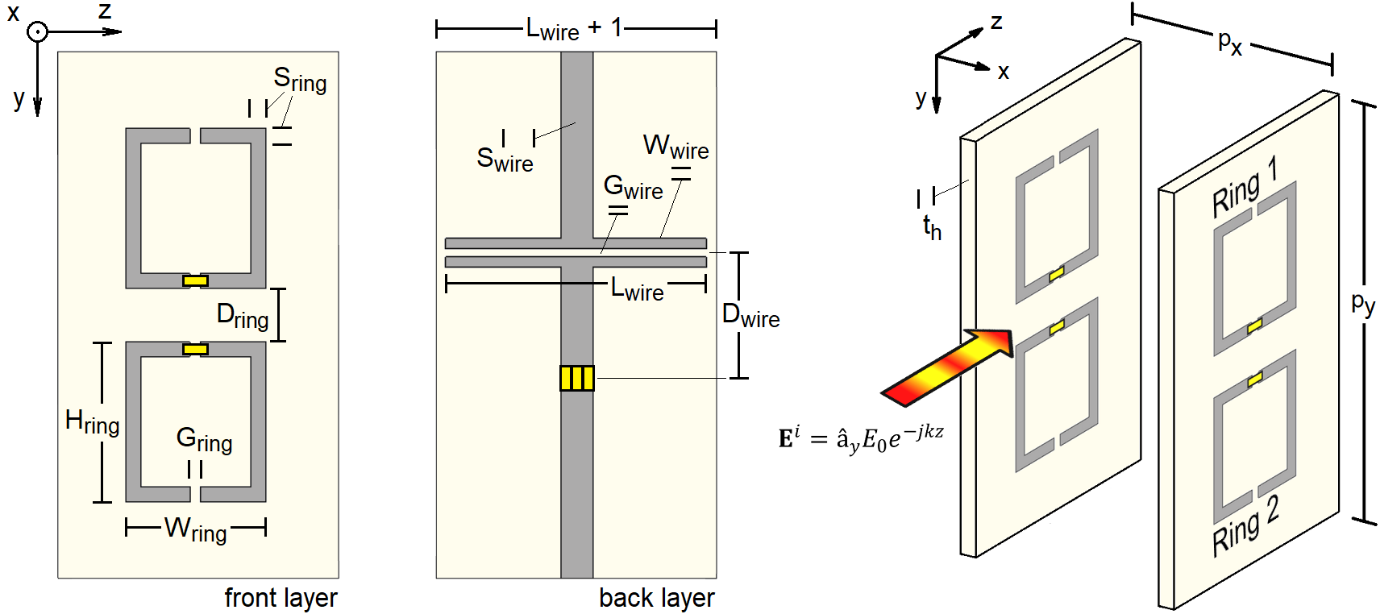
**

**Supplementary Fig. 5.** Metasurface superstrate unit cell: a couple of symmetric split rings in one layer, and a cut wire in the other; varactors are in yellow. Our simulations set an impinging wave of electric field $\mathbf{E}^{\boldsymbol{i}}$.

In the recently presented cell^33^, rings 1 and 2 are symmetric. To work properly in the desired bands B1–B4, such unit cell was adjusted as summarized in Supplementary Table 3 and rings 1 and 2 became asymmetric. The employed substrate has thickness $t_{h}=1.27 mm$, relative permittivity $\varepsilon_{r}=10.5$, and loss tangent $\tan\delta=0.0023$.

**Supplementary Table 3.** Parameters of the metasurface superstrate unit cell

| **Symbol** | **Description** | **Value** |
| --- | --- | --- |
| $\varepsilon_{r}$ | substrate relative permittivity | $10.5$ |
| $D_{ring}$ | distance between rings | $2.92 mm$ |
| $D_{wire}$ | distance between the capacitor-like structure and varactors | $6.82 mm$ |
| $G_{ring_{1}}$ | gap in the ring 1 | $0.59 mm$ |
| $G_{ring_{2}}$ | gap in the ring 2 | $0.59 mm$ |
| $G_{wire}$ | gap in the capacitor-like structure | $0.20 mm$ |
| $H_{ring_{1}}$ | height of the ring 1 | $8.29 mm$ |
| $H_{ring_{2}}$ | height of the ring 2 | $8.14 mm$ |
| $L_{wire}$ | length of the capacitor-like structure | $16.48 mm$ |
| $p_{x}$ | periodicity in the $x$ dimension | $33.27 mm$ |
| $p_{y}$ | periodicity in the $y$ dimension | $28.66 mm$ |
| $S_{ring_{1}}$ | width of the strip of the ring 1 | $0.83 mm$ |
| $S_{ring_{2}}$ | width of the strip of the ring 2 | $0.83 mm$ |
| $S_{wire}$ | width of the wire strip | $1.76 mm$ |
| $W_{ring_{1}}$ | width of the ring 1 | $8.78 mm$ |
| $W_{ring_{2}}$ | width of the ring 2 | $10.24 mm$ |
| $W_{wire}$ | width of the capacitor-like structure | $0.68 mm$ |
| $\tan\delta$ | substrate loss tangent | $0.0023$ |
| $t_{h}$ | substrate thickness | $1.27 mm$ |

The transmission coefficient $\tau_{sup}$ was calculated with the transient solver of CST Microwave Studio. The unit cell was oriented as in Supplementary Fig. 5. The boundary conditions on the directions $\pmâ_{x}$, $\pmâ_{y}$, and $\pmâ_{z}$ were respectively set as PMC, PEC, and open, mimicking thereby an infinite array a TEM wave with electric field $\mathbf{E}^{i}=\mathbf{â}_{y}E_{0}e^{-jkz}$ was normally incident upon. Supplementary Fig. 6 shows the simulated transmission coefficient magnitude $\left| \tau_{sup} \right|$ and phase $\varphi_{\tau_{sup}}$ for two values of capacitance ($C=0.03 \mathrm{pF}$ and $C=0.20 \mathrm{pF}$).

| 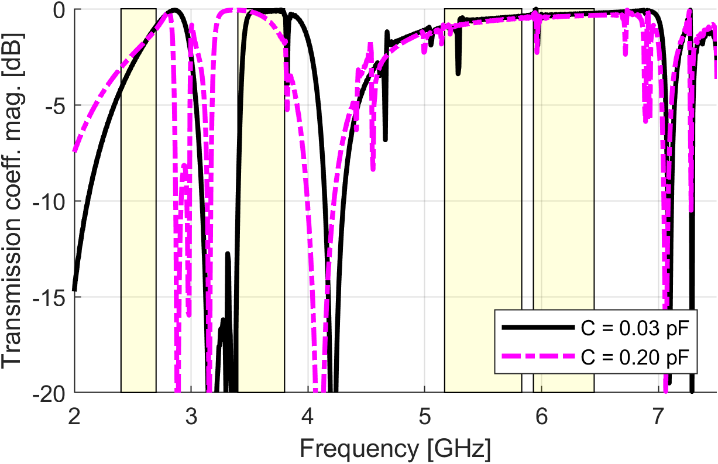 | 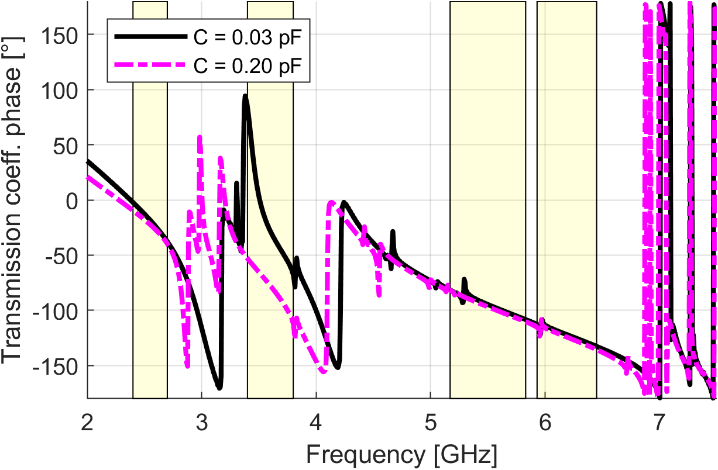 |
| --- | --- |
| (a) | (b) |
| **Supplementary Fig. 6.** Transmission coefficient of the initial unit cell for $C=0.03 pF$  : (a) magnitude $\left\vert\tau_{sup} \right\vert$; and (b) phase $\varphi_{\tau_{sup}}$. The operating frequency bands of interest B1–B4 are highlighted. | |

The maximum transmission phase variation in the B2 band is $\Delta{\varphi_{\tau}}_{sup}=148^{\circ}$, which indicates that this unit cell can steer electromagnetic beams at this frequency band if adjacent unit cells impose a gradual phase shift to transmitted waves according to the generalized law of refraction^12^. At $2.40 \mathrm{GHz}$, the maximum transmission phase variation is $\Delta{\varphi_{\tau}}_{sup}=8^{\circ}$, which means that a negligible beam steering can occur in the Wi-Fi 2.4 standard when the capacitance $C$ is changed. In the B3 and B4 bands, resonance-like phenomena dependent on the value of the capacitance $C$ are seen. These phenomena are present in the transmission phase ${\varphi_{\tau}}_{sup}$ too, indicating that the Wi-Fi 5/6E standards may also be affected when the capacitance $C$ is changed. Supplementary Fig. 7 shows the reflection coefficient magnitude $\left| \Gamma_{sup} \right|$ and phase $\varphi_{\Gamma_{sup}}$ of the unit cell for varactor capacitance values of $C=0.03 \mathrm{pF}$ and $C=0.20 \mathrm{pF}$.

| 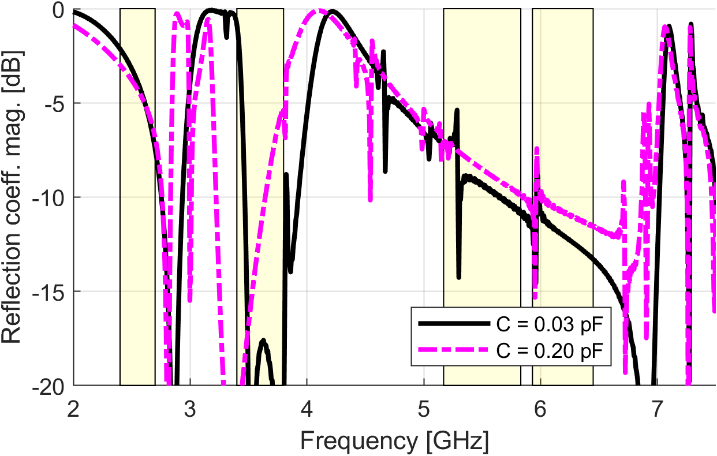 | 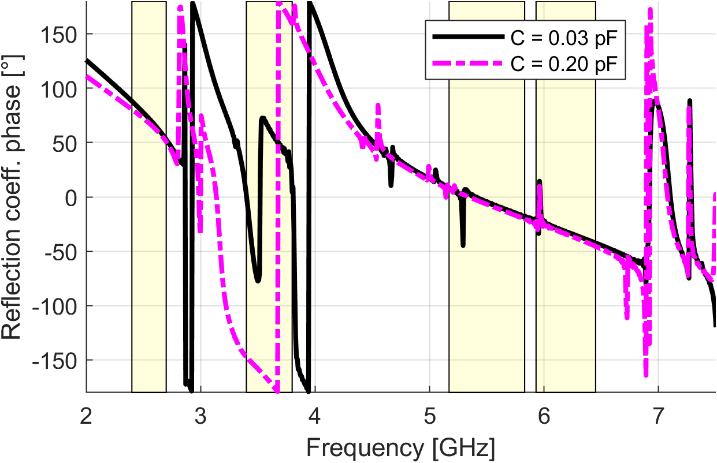 |
| --- | --- |
| (a) | (b) |
| **Supplementary Fig. 7.** Reflection coefficient of the initial unit cell for $C=0.03 pF$  : (a) magnitude $\left\vert\tau_{sup} \right\vert$; and (b) phase $\varphi_{\tau_{sup}}$. The operating frequency bands of interest B1–B4 are highlighted. | |

Magnitudes around $-4.0$, $-17.0$, $-8.9$, and $-11.4 \mathrm{dB}$ are respectively seen in the center frequencies of the B1–B4 bands. Concerning the reflection coefficient phase $\varphi_{\Gamma_{sup}}$, the B2 band presents a variation of $\Delta\varphi_{\Gamma_{sup}}=147^{\circ}$, at $3.40 \mathrm{GHz}$, while in the B1 band a $\Delta\varphi_{\Gamma_{sup}}$ of $8^{\circ}$ is seen at $2.40 \mathrm{GHz}$. Resonance-like phenomena dependent on the value of the capacitance $C$ are seen again in the B3 and B4 bands in both magnitude and phase graphs.

One could propose that the results of this unit cell cannot be considered ideal for the purpose of this work because of the resonance-like phenomena in the bands B3 and B4. However, these results were obtained for conditions that emulate an infinite array upon which a TEM wave is normally incident. It is expected that, when a finite-array metasurface superstrate based on this cell is put in the near-field region of the antenna, the transmission bands and other phenomena seen in Supplementary Fig. 6 shift in frequency. Thus, it was not worth optimizing these results at this stage.

**Supplementary Note 3. Application of the methodology to the proposed design**

The reflection coefficient phase of the AMC used in this work is less versatile in the joint B3+B4 band, where it presents a reflection phase asymptotically going to $\varphi_{\Gamma_{refl}}\left( f \right)=-180^{\circ}$, than in the B1 and B2 bands, where it presents easily controllable resonances. Hence, we accomplish the proposed methodology as:

1. From the parametric study of the employed AMC unit cell presented in this recent work^32^ we observe typical values for the reflection coefficient phase $\varphi_{\Gamma_{refl}}$ around $-130^{\circ}$ at $f_{l}=5.17 GHz$, and around $-160^{\circ}$ at $f_{h}=6.45 \mathrm{GHz}$; using (4), we have:

| $\frac{c}{5.17\times{10}^{9}}\left( \frac{-130^{\circ}}{4\pi}+\frac{1}{3} \right)$ | $<$ | $h_{refl}$ | $\boldsymbol{<}$ | $\frac{\boldsymbol{c}}{\boldsymbol{6.45\times}\boldsymbol{10}^{\boldsymbol{9}}}\left( \frac{\boldsymbol{-1}\boldsymbol{6}\boldsymbol{0^{\circ}}}{\boldsymbol{4}\boldsymbol{\pi}}\boldsymbol{+}\frac{\boldsymbol{2}}{\boldsymbol{3}} \right)$ |  |
| --- | --- | --- | --- | --- | --- |
| $8.9 mm$ | $<$ | $h_{refl}$ | $\boldsymbol{<}$ | $\boldsymbol{20.7 mm}$ | (S.1) |

1. Then, we evaluate the range of reflection phases $\varphi_{\Gamma_{refl}}\in\left[ \varphi_{\Gamma_{refl_{1}}},\varphi_{\Gamma_{refl_{2}}} \right]$ for the B1 and B2 bands considering the condition I and $8.9 mm<h_{refl}<20.7 mm$; from (5), for the B1 band, we have:

| $-\frac{2\pi}{3}+\frac{4\pi\times2.55\times{10}^{9}}{c}0.0089$ | $<$ | $\varphi_{\Gamma_{refl}}$ | $\boldsymbol{<}$ | $\boldsymbol{+}\frac{\boldsymbol{2}\boldsymbol{\pi}}{\boldsymbol{3}}\boldsymbol{+}\frac{\boldsymbol{4}\boldsymbol{\pi}\boldsymbol{\times2.55\times}\boldsymbol{10}^{\boldsymbol{9}}}{\boldsymbol{c}}\boldsymbol{0.0207}$ |  |
| --- | --- | --- | --- | --- | --- |
| $-65.7^{\circ}$ | $<$ | $\varphi_{\Gamma_{refl}}$ | $\boldsymbol{<}$ | $\boldsymbol{+246.5^{\circ}}$ | (S.2) |

For the B2 band, we have:

| $-\frac{2\pi}{3}+\frac{4\pi\times3.60\times{10}^{9}}{c}0.0089$ | $<$ | $\varphi_{\Gamma_{refl}}$ | $\boldsymbol{<}$ | $\boldsymbol{+}\frac{\boldsymbol{2}\boldsymbol{\pi}}{\boldsymbol{3}}\boldsymbol{+}\frac{\boldsymbol{4}\boldsymbol{\pi}\boldsymbol{\times3.60\times}\boldsymbol{10}^{\boldsymbol{9}}}{\boldsymbol{c}}\boldsymbol{0.0207}$ |  |
| --- | --- | --- | --- | --- | --- |
| $-43.4^{\circ}$ | $<$ | $\varphi_{\Gamma_{refl}}$ | $\boldsymbol{<}$ | $\boldsymbol{+2}\boldsymbol{9}\boldsymbol{8.6^{\circ}}$ | (S.3) |

1. Now that we have the possible values for $\varphi_{\Gamma_{refl}}$ in each band, we may evaluate the possible values for the spacing $h_{FP}\in\left[ h_{FP_{1}},h_{FP_{2}} \right]$; according to Supplementary Fig. 7b, the reflection phase $\varphi_{\Gamma_{sup}}$ of the superstrate for the center frequency of the B1 band ($f_{c}=2.55 GHz$) is $+68^{\circ}$; using (6) and $N=0, 1, 2$, we have:

| $0.4 mm$ | $<$ | $h_{FP}$ | $\boldsymbol{<}$ | $\boldsymbol{51.4 mm}$ | (S.4) |
| --- | --- | --- | --- | --- | --- |
| $59.2 mm$ | $<$ | $h_{FP}$ | $\boldsymbol{<}$ | $\boldsymbol{110.2 mm}$ | (S.5) |
| $118.0 mm$ | $<$ | $h_{FP}$ | $\boldsymbol{<}$ | $\boldsymbol{169.0 mm}$ | (S.6) |

At the center frequency of the B2 band ($f_{c}=3.60 GHz$), $\varphi_{\Gamma_{sup}}$ is around $-120^{\circ}$; using (6), and $N=0, 1, 2$:

| $-18.9 mm$ | $<$ | $h_{FP}$ | $\boldsymbol{<}$ | $\boldsymbol{20.7 mm}$ | (S.7) |
| --- | --- | --- | --- | --- | --- |
| $22.8 mm$ | $<$ | $h_{FP}$ | $\boldsymbol{<}$ | $\boldsymbol{62.3 mm}$ | (S.8) |
| $64.4 mm$ | $<$ | $h_{FP}$ | $\boldsymbol{<}$ | $\boldsymbol{104.0 mm}$ | (S.9) |

For the center frequency of the joint B3+B4 band ($f_{c}=5.81 GHz$), $\varphi_{\Gamma_{sup}}=-20^{\circ}$; then:

| $-12.9 mm$ | $<$ | $h_{FP}$ | $\boldsymbol{<}$ | $\boldsymbol{-10.8 mm}$ | (S.10) |
| --- | --- | --- | --- | --- | --- |
| $12.9 mm$ | $<$ | $h_{FP}$ | $\boldsymbol{<}$ | $\boldsymbol{15.1 mm}$ | (S.11) |
| $38.7 mm$ | $<$ | $h_{FP}$ | $\boldsymbol{<}$ | $\boldsymbol{40.9 mm}$ | (S.12) |

We define the spacing between the superstrate and AMC as $12.9 mm<h_{refl}<15.1 mm$, which is the intersection of (S.4), (S.7) and (S.11); also, this range meets condition III since $h_{FB}>h_{refl_{1}}$.

1. Following, for the B1 and B2 bands, we define a reflection phase $\varphi_{\Gamma_{refl}}$ that respects condition II given the defined range $12.9 mm<h_{FP}<15.1 mm$; according to Supplementary Fig. 7b, the reflection phase of the superstrate for the center frequency of the B1 band ($f_{c}=2.55 GHz$) is $\varphi_{\Gamma_{sup}}=+68^{\circ}$; using (7) and $N=0$:

| $\frac{4\pi\times2.55\times{10}^{9}}{c}0.0129-68^{\circ}$ | $<$ | $\varphi_{\Gamma_{refl}}$ | $\boldsymbol{<}$ | $\frac{\boldsymbol{4}\boldsymbol{\pi}\boldsymbol{\times2.55\times}\boldsymbol{10}^{\boldsymbol{9}}}{\boldsymbol{c}}\boldsymbol{0.0151}\boldsymbol{-68^{\circ}}$ |  |
| --- | --- | --- | --- | --- | --- |
| $+11.0^{\circ}$ | $<$ | $\varphi_{\Gamma_{refl}}$ | $\boldsymbol{<}$ | $\boldsymbol{+24.2^{\circ}}$ | (S.13) |

With this range of $\varphi_{\Gamma_{refl}}$ for the B1 band and from condition I:

| $\frac{c}{4\pi\times2.55\times{10}^{9}}\left( +11.0^{\circ}-\frac{2\pi}{3} \right)$ | $<$ | $h_{refl}$ | $\boldsymbol{<}$ | $\frac{\boldsymbol{c}}{\boldsymbol{4}\boldsymbol{\pi}\boldsymbol{\times2.55\times}\boldsymbol{10}^{\boldsymbol{9}}}\left( \boldsymbol{+24.2^{\circ}+}\frac{\boldsymbol{2}\boldsymbol{\pi}}{\boldsymbol{3}} \right)$ |  |
| --- | --- | --- | --- | --- | --- |
| $-17.8 mm$ | $<$ | $h_{refl}$ | $\boldsymbol{<}$ | $\boldsymbol{2}\boldsymbol{3}\boldsymbol{.6 mm}$ | (S.14) |

For the B2, according to Supplementary Fig. 7b, the reflection phase of the superstrate for the center frequency ($f_{c}=3.60 GHz$) is $\varphi_{\Gamma_{sup}}$ around $-120^{\circ}$; using (7) and considering $N=-1$:

| $\frac{4\pi\times3.60\times{10}^{9}}{c}0.0129-240^{\circ}$ | $<$ | $\varphi_{\Gamma_{refl}}$ | $\boldsymbol{<}$ | $\frac{\boldsymbol{4}\boldsymbol{\pi}\boldsymbol{\times3.60\times}\boldsymbol{10}^{\boldsymbol{9}}}{\boldsymbol{c}}\boldsymbol{0.0151}\boldsymbol{-240^{\circ}}$ |  |
| --- | --- | --- | --- | --- | --- |
| $-128.5^{\circ}$ | $<$ | $\varphi_{\Gamma_{refl}}$ | $\boldsymbol{<}$ | $\boldsymbol{-109.9^{\circ}}$ | (S.15) |

With this range of $\varphi_{\Gamma_{refl}}$ for the B2 and from condition I:

| $\frac{c}{4\pi\times3.60\times{10}^{9}}\left( +231.5^{\circ}-\frac{2\pi}{3} \right)$ | $<$ | $h_{refl}$ | $\boldsymbol{<}$ | $\frac{\boldsymbol{c}}{\boldsymbol{4}\boldsymbol{\pi}\boldsymbol{\times3.60\times}\boldsymbol{10}^{\boldsymbol{9}}}\left( \boldsymbol{+250.1^{\circ}+}\frac{\boldsymbol{2}\boldsymbol{\pi}}{\boldsymbol{3}} \right)$ |  |
| --- | --- | --- | --- | --- | --- |
| $12.9 mm$ | $<$ | $h_{refl}$ | $\boldsymbol{<}$ | $\boldsymbol{42.8 mm}$ | (S.16) |

The range associated to the B3+B4 joint band was already evaluated in (S.1). We take the value $h_{refl}=12.9 mm$ from the intersection of (S.1), (S.14) and (S.16). As the final value for the spacing between superstrate and reflector, we take $h_{FP}=15.1 mm$. For the reflection phase $\varphi_{\Gamma_{refl}}$, we respectively take the following values for the center frequencies of the B1, B2 and joint B3+B4 bands: $+24.2^{\circ}$, $-128.5^{\circ}$, $-160.0^{\circ}$. These phase values were respectively chosen in the ranges (S.13), (S.15), and from the step a) because, among those that can meet condition II, they minimize $\left| \varphi_{\Gamma_{refl}}-2kh_{refl} \right|$ optimizing thereby the gain in the broadside direction. Table 1 in the main text summarizes the chosen parameters. Such parameters serve as a starting point for the design of the complete device, which should be optimized through full-wave simulations which take into account the actual incidence of waves over the AMC and the superstrate in the final design.

**Supplementary Note 4. Optimization of the Huygens metasurface and the double-square AMC**

In main-text Fig. 3a, the drops of gain in the middle of the B1 band and second half of joint B3+B4 bands for $h_{FP}=15.1 mm$ require adjustments. The strong variations of broadside realized gain as a function of $h_{FP}$ denounce that the superstrate is not transmitting well in these frequency ranges. At this point, we believed that, due to differences in the conditions of incidence of waves between the simulation of the unit cell and the complete design, the higher frequency limit around $7.00 \mathrm{GHz}$ seen in the transmission coefficient curves in Supplementary Fig. 6 appeared at a lower frequency in the complete device and was constraining the B4 as seen in the black curve in main-text Fig. 3a. Our goal was to substantially shift this limit upward in frequency. We were also interested in enlarging the first transmission band, so that the drop of gain in the B1 was mitigated. To handle this problem, a study on the behavior of the Huygens metasurface unit cell was done by varying each parameter listed on Supplementary Table 3 one by one. We identified the most sensitive parameters and optimized the structure to shift the drops in gain observed in Fig. 3a. To take into account the actual incidence of waves in the complete device, all the adjustments were made one by one by taking the broadside realized gain as a reference. The parametric study on the transmission coefficient of the unit cell was only useful to indicate which parameter should be increased or decreased. Supplementary Table 4 lists the definitive parameters of the Huygens unit cell after the optimization. The parameters studied above presented the largest changes (bold). The others had a slight adjustment.

**Supplementary Table 4.** Parameters of the metasurface superstrate unit cell

| Symbol | Description | Initial Cell | Definitive Cell |
| --- | --- | --- | --- |
| $\varepsilon_{r}$ | substrate relative permittivity | $10.5$ | $10.5$ |
| $D_{ring}$ | distance between rings | $2.92 mm$ | $4.00 mm$ |
| $D_{wire}$ | distance between the capacitor-like structure and varactors | $6.82 mm$ | $6.00 mm$ |
| $G_{ring_{1}}$ | gap in the ring 1 | $0.59 mm$ | $0.59 mm$ |
| $G_{ring_{2}}$ | gap in the ring 2 | $0.59 mm$ | $0.59 mm$ |
| $G_{wire}$ | gap in the capacitor-like structure | $0.20 mm$ | $0.20 mm$ |
| $H_{ring_{1}}$ | height of the ring 1 | $8.29 mm$ | $9.00 mm$ |
| $H_{ring_{2}}$ | height of the ring 2 | $8.14 mm$ | $6.50 mm$ |
| $L_{wire}$ | length of the capacitor-like structure | $16.48 mm$ | $11.25 mm$ |
| $p_{x}$ | periodicity in the $x$ dimension | $33.27 mm$ | $25.00 mm$ |
| $p_{y}$ | periodicity in the $y$ dimension | $28.66 mm$ | $30.00 mm$ |
| $S_{ring_{1}}$ | width of the strip of the ring 1 | $0.83 mm$ | $0.85 mm$ |
| $S_{ring_{2}}$ | width of the strip of the ring 2 | $0.83 mm$ | $0.85 mm$ |
| $S_{wire}$ | width of the wire strip | $1.76 mm$ | $0.20 mm$ |
| $W_{ring_{1}}$ | width of the ring 1 | $8.78 mm$ | $9.00 mm$ |
| $W_{ring_{2}}$ | width of the ring 2 | $10.24 mm$ | $11.00 mm$ |
| $W_{wire}$ | width of the capacitor-like structure | $0.68 mm$ | $0.20 mm$ |
| $\tan\delta$ | substrate loss tangent | $0.0023$ | $0.0023$ |
| $t_{h}$ | substrate thickness | $1.27 mm$ | $1.27 mm$ |

Supplementary Fig. 8 shows the broadside realized gain for the complete device with a metasurface superstrate based on the definitive unit cell described in Supplementary Table 4.

**
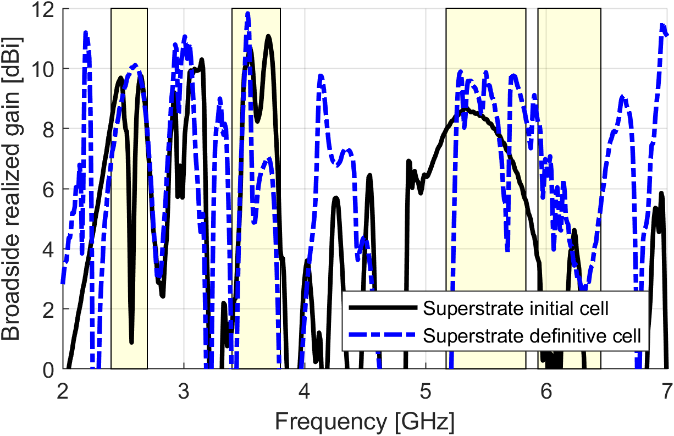
**

**Supplementary Fig. 8.** Simulated broadside realized gain for the complete device with a Huygens metasurface superstrate

based on the definitive unit cell of Supplementary Table 4.

It is apparent that the definitive unit cell solves the problem of the gain drop in the band B1. Besides that, the higher frequency limit of operation in the B4 shifted upward. On the other hand, in the beginning of the B3, the broadside realized gain is not in a good level and a drop of gain appeared at $5.65 \mathrm{GHz}$, indicating that the resonance-like phenomena that were seen around $4.50$ and $5.25 \mathrm{GHz}$ in the transmission coefficient curves of the unit cell indeed appeared in this band. Lastly, in the second half of the band B2, the gain drops from levels around $9.5 \mathrm{dBi}$ to levels around $6.0 \mathrm{dBi}$. Making a new attempt to optimize the superstrate would consume a remarkable time in the development activity. Hence, in what follows, we show an optimization of the AMC and its spacing $h_{refl}$ with the radiating element.

We also identified the most sensitive parameters of the AMC and optimized it to enhance the gain observed in Supplementary Fig. 8. After sweeping the periodicity $p$ and the spacing $h_{refl}$ in specific ranges, we concluded that a periodicity $p=18.3 mm$ and a spacing $h_{refl}=11.0 mm$ improved the performance of the complete device in relation to what is seen in Supplementary Fig. 8. Optimizing the performance through the periodicity is particularly interesting as a means of achieving a better compactness in the final device as well. We also verified that adjusting the inner gap $g_{2}$ and the strip between gaps $s$ to $0.20 mm$ does not compromise the device’s performance, while it facilitates the fabrication process. Supplementary Table 5 lists the parameters of the definitive AMC unit cell.

**Supplementary Table 5.** Evolution of parameters from the initial to the definitive AMC unit cell

| Symbol | Description | Initial cell | Definitive cell |
| --- | --- | --- | --- |
| $\varepsilon_{r}$ | substrate relative permittivity | $2.34$ | $2.34$ |
| $g_{1}$ | outer gap | $0.65 mm$ | $0.65 mm$ |
| $g_{2}$ | inner gap | $0.10 mm$ | $0.20 mm$ |
| $p$ | periodicity | $20.5 mm$ | $18.3 mm$ |
| $s$ | strip between gaps | $0.10 mm$ | $0.20 mm$ |
| $\tan\delta$ | substrate loss tangent | $0.0013$ | $0.0013$ |
| $t_{h}$ | substrate thickness | $4.9 mm$ | $4.9 mm$ |

Supplementary Fig. 9 shows the broadside realized gain of the complete device with the definitive cells in both the Huygens metasurface and the AMC.

**
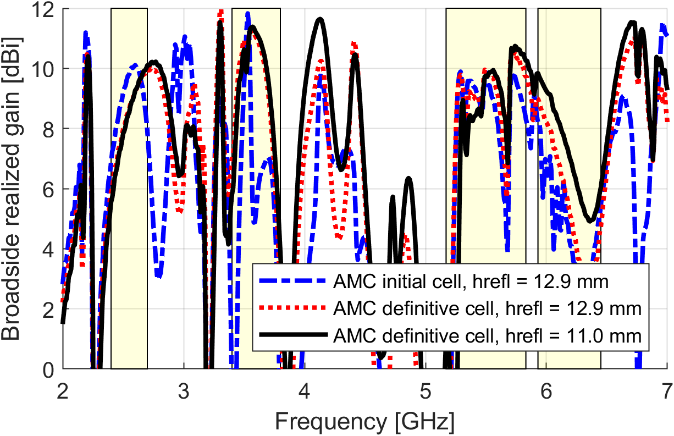
**

**Supplementary Fig. 9.** Simulated broadside realized gain the complete device with an AMC based on the definitive unit cell of Supplementary Table 5.

The broadside realized gain was substantially improved in the bands B2 and B4 when the definitive AMC was placed at a distance $h_{refl}=11.0 mm$ from the adjusted bow-tie. Conversely, in the beginning of both bands B1 and B3, the rise of gain was shifted upward in frequency. At this point, we decided that further optimizations in the device should take place after the insertion of the varactor feeding lines, the superstrate mechanical support, the foam, and the balun, considering that the insertion of these elements may have an impact on the performance.

**Supplementary Note 5. Insertion of final elements**

To provide the desired values of capacitance $C$, the varactors of each column of the Huygens metasurface should be biased by a specific voltage through a feeding line. Aiming at not changing the performance in relation to Supplementary Fig. 9, the feeding lines are sectioned in small pieces much smaller than the wavelength at the highest frequency of operation ($6.45 \mathrm{GHz}$). Resistors are used to connect these pieces and the original metal parts of the Huygens cells. Copper wires with $0.2 \mathrm{mm}$ of diameter are used to connect both layers of the cells. The dimensions of the feeding lines are indicated in Supplementary Fig. 10. Resistors are in green.


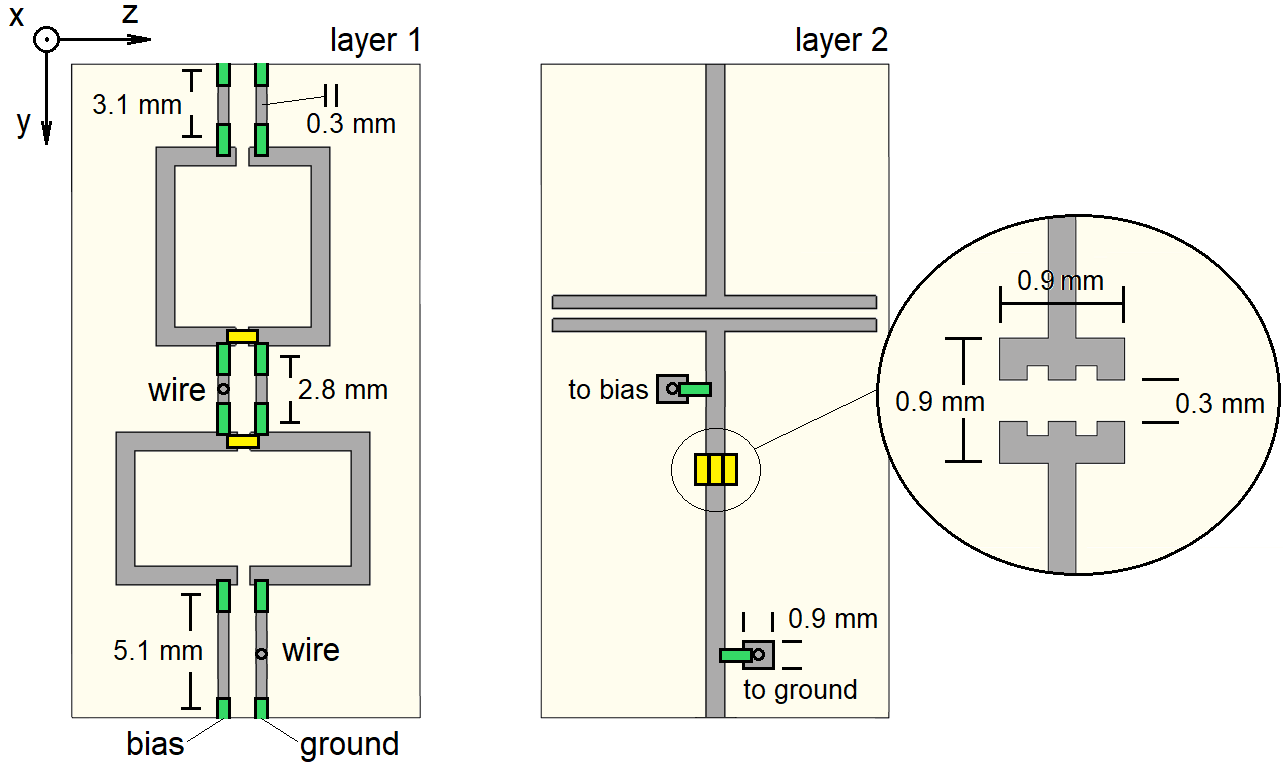


**Supplementary Fig. 10.** Huygens unit cell with feeding lines

; resistors are in green.

The inset in Supplementary Fig. 10 details the pads over which the three varactors were mounted on layer 2. The resistors were dimensioned to a value of $8.2 kΩ$. This value was large enough to mitigate the flow of currents through the feeding lines. The MACOM MAVR-011020-1411 datasheet reports a maximum reverse leakage current of $100 \mathrm{nA}$. The value of $8.2 kΩ$ was also small enough to avoid a significant voltage drop due to these reverse leakage currents, which would make varactors of a same column be subjected to different bias voltages.

It is worth mentioning that the complete device was slightly asymmetric from this point on since the wires and pads in the unit cell causes an asymmetry in relation to the $xy$-plane. In the complete device, instead of adopting two couples of mirrored columns of this unit cell, we chose to adopt four identical columns in order to make them interchangeable or easily substituted in the case of damages after the fabrication. Supplementary Fig. 11 shows the broadside realized gain of the complete device after the insertion of the feeding lines.


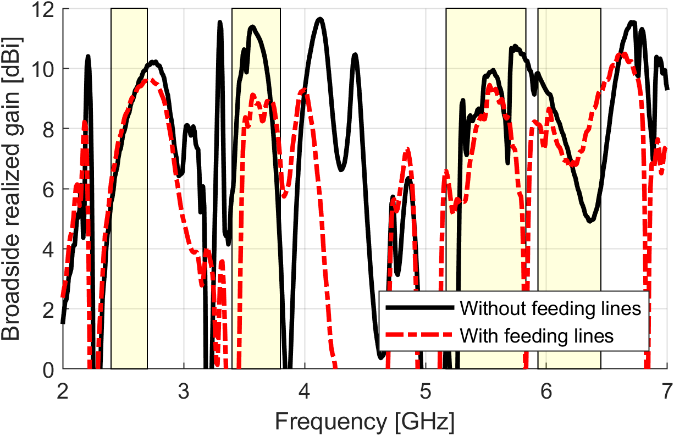


**Supplementary Fig. 11.** Simulated broadside realized gain the complete device after the insertion of the feeding lines.

The feeding lines impacted the four B1–B4 bands but did not compromise the performance. In the B1, the peak value for the broadside realized gain dropped from $10.1$ to $9.6 dBi$. In the B2, it dropped from $11.3$ to $8.8 dBi$. In the B3, the insertion of the feeding lines caused the rise of gain to be shifted downward in frequency, and the band became totally covered with at least a moderate gain level ($\geq5.0 dBi$). An improvement was also seen in the B4, where the minimum value raised from $4.9$ to $6.7 dBi$. The insertion of the feeding lines caused the appearance of a drop of gain in the second half of the B3. In our simulations, the frequency of this drop has shown to be dependent on the values of the varactor capacitances $C$. In an attempt to shift this drop to the region between the bands B3 and B4, we used $0.03$, $0.08$, $0.08$, and $0.03 pF$ as the respective values for the capacitances $C$ for the metasurface columns $1$–$4$ in the simulation seen in Supplementary Fig. 11. These values were assumed hereafter.

Next, the mechanical support of the superstrate was considered. Made on an Arlon DiClad 870 substrate whose thickness is $2.45 mm$, relative permittivity $\varepsilon_{r}=2.34$, and $\tan\delta=0.0013$, the support consisted of four couple of pieces, each piece with an outer and an inner layer. The strips in the outer layers transmit to each columns the bias voltage from a generator connected in the back of the device. The strips in the inner layers connect the columns to the AMC ground plane, where the DC ground of the voltage generators should be connected. Supplementary Fig. 12 illustrates the disposition of the mechanical support in the complete device.


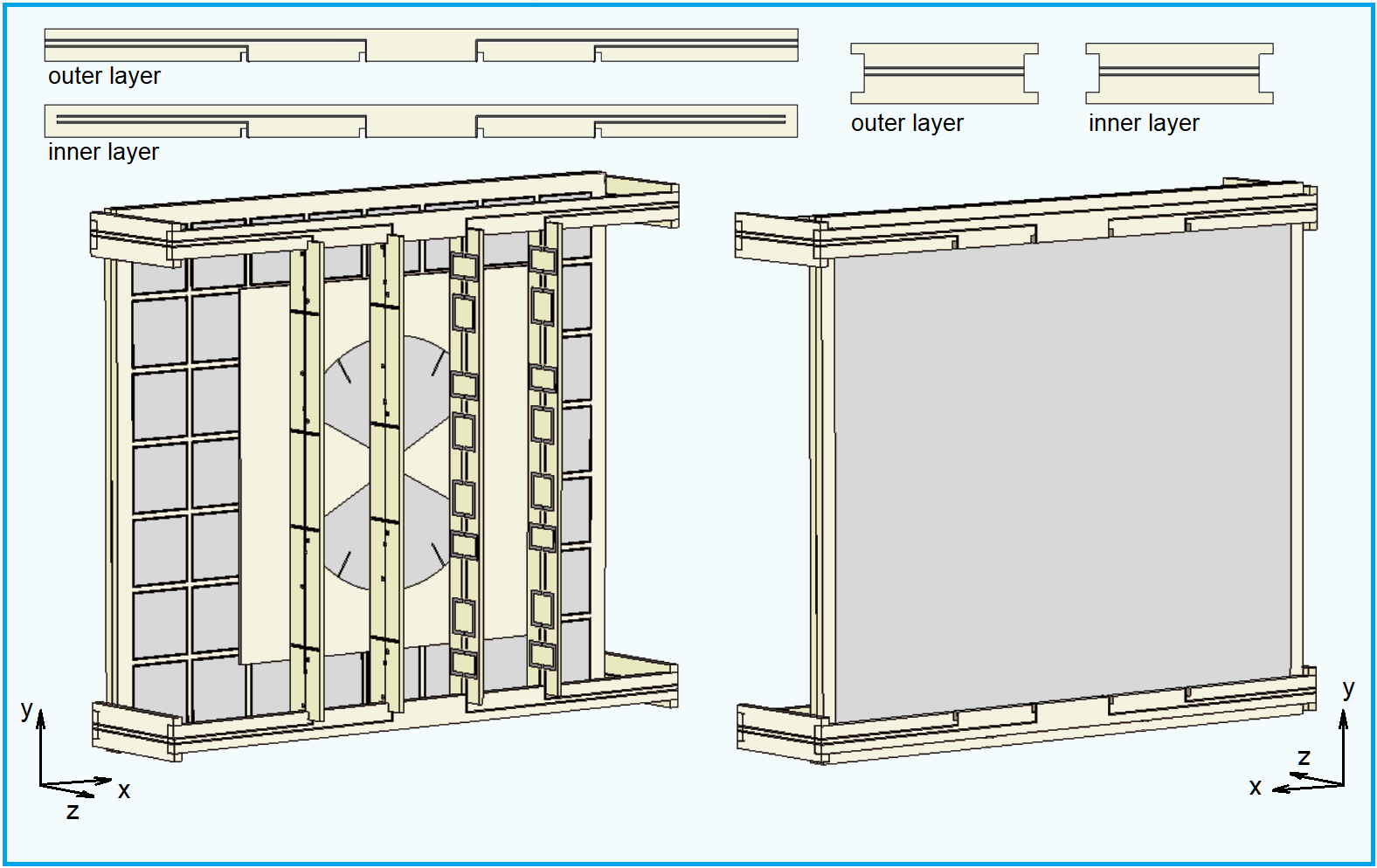


**Supplementary Fig. 12.** Insertion of the superstrate mechanical support

: the inner layers connect the AMC ground plane to the ground of the varactors; the outer layers transmit the bias voltages to the varactors.

Supplementary Fig. 13 shows the simulated broadside realized gain of the complete device after the insertion of the superstrate mechanical support.


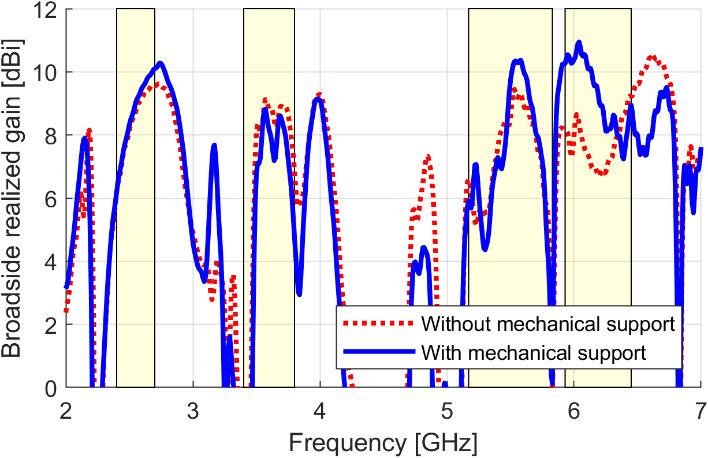


**Supplementary Fig. 13.** Simulated broadside realized gain the complete device after the mechanical support insertion.

The insertion of the mechanical support also impacted the four B1–B4 bands, but again not compromising performance. In the B1, the peak value for the broadside realized gain raised from $9.6$ to $10.1 dBi$. In the B2, it dropped from $8.9$ to $8.5 dBi$. In the B3, the mechanical support increased the ripple, making the peak gain raise from $9.2$ to $10.3 dBi$, and the minimum gain drop from $5.0$ to $3.9 dBi$. In the B4, the peak of broadside realized gain raised from $9.2$ to $10.8 dBi$.

In what follows, the foam bricks, the screws, and the balun were inserted. An exponential taper balun presents a $3:1$-impedance ratio and allows the feeding of the structure by a $50$-$Ω$ SMA connector, which is terminated by a waveguide port in all the simulations hereafter. This balun was etched on an Arlon DiClad 880 substrate (thickness $0.76 mm$, relative permittivity $\varepsilon_{r}=2.20$, and $\tan\delta=0.0009$). Twelve $3 mm$-diameter Teflon screws were used to fix two sheets of Arlon DiClad 870 that were used in the AMC. For mechanical stability, a foam brick (relative permittivity $\varepsilon_{r}=1.1$, dissipation factor $\tan\delta=0.005$) surrounded the balun in the back of the device. Two other bricks were placed between the adjusted bow-tie and the AMC, ensuring the appropriate spacing $h_{refl}=11 mm$ between them. Main-text Fig. 4 shows the final design in CST Microwave Studio. Supplementary Fig. 14 shows the simulated broadside realized gain of the complete device after the insertion of the foam bricks, the screws, and the balun.


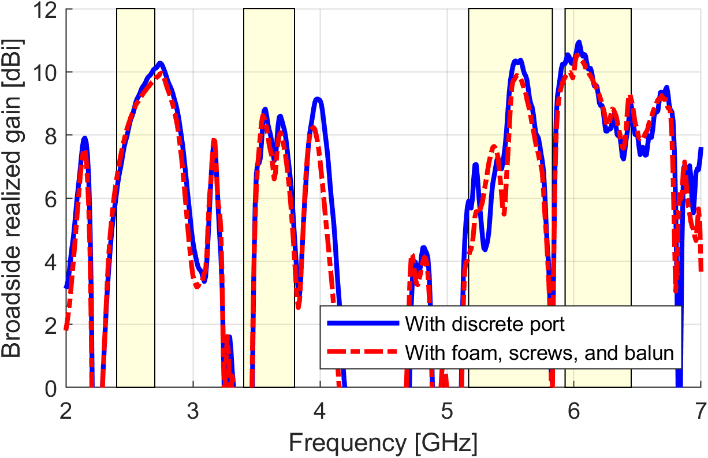


**Supplementary Fig. 14.** Simulated broadside realized gain the complete device after the insertion of the foam bricks

, the screws, and the balun.

Almost no impact is seen throughout all the B1–B4 bands due to the insertion of the foam bricks, screws, and balun.

**Supplementary Note 6. Assembling details**

The prototype was etched with an LPKF ProtoLaser S4 machine. For the grooved bow-tie, an Arlon DiClad 880 substrate (thickness $0.76 \mathrm{mm}$, relative permittivity $\varepsilon_{r}=2.20$, and loss tangent $\tan\delta=0.0009$) was used. For the AMC, two sheets of Arlon DiClad 870 substrate whose thickness is $2.45 mm$, relative permittivity $\varepsilon_{r}=2.34$, and $\tan\delta=0.0013$ were fixed with Teflon screws to provide the final thickness of $4.90 \mathrm{mm}$. The Huygens metasurface was etched on an Arlon AD1000 laminate (thickness $1.27 \mathrm{mm}$, relative permittivity $\varepsilon_{r}=10.5$, and the loss tangent $\tan\delta=0.0023$).

The in-house assembling of the Huygens elements included a difficult stage of manually welding 80 varactors (model MACOM MAVR-011020-1411) and 184 resistors (model MULTICOMP E24 series, $8.2 kΩ$) with the aid of an electronic microscope. The final prototype, including the grooved rounded-edge bow-tie and the AMC, is shown in main-text Fig. 5. A $0.2$-$\mathrm{mm}$ copper wire was used to connect the edges of the metallic strips in the mechanical support.

Two dual-output DC power supplies model Hewlett Packard E3620A were used in the feeding of the varactors. The power supplies were positioned outside the anechoic chamber and connected to the feeding lines of the device through $0.2$-$\mathrm{mm}$ copper wires. The bias voltages were optimized to perform the beam-steering function in the range $3.50$–$3.65 \mathrm{GHz}$. Supplementary Table 6 summarizes the values for five beam-steering states. Imperfections in the fabricated device required the bias voltages related to states $-1$ and $+1$ not to be symmetric.

**Supplementary Table 6.** Bias voltages for the five beam-steering states

| **State** | **Column 1** | **Column 2** | **Column 3** | **Column 4** |
| --- | --- | --- | --- | --- |
| $\boldsymbol{-2}$ | $0.0 V$ | $0.0 V$ | $9.0 V$ | $13.0 V$ |
| $\boldsymbol{-1}$ | $1.7 V$ | $1.6 V$ | $3.0 V$ | $10.0 V$ |
| $\boldsymbol{0}$ | $3.3 V$ | $1.5 V$ | $1.5 V$ | $3.3 V$ |
| $\boldsymbol{+1}$ | $13.0 V$ | $2.9 V$ | $1.0 V$ | $1.6 V$ |
| $\boldsymbol{+2}$ | $13.0 V$ | $9.0 V$ | $0.0 V$ | $0.0 V$ |

| 12345678910111213141516171819202122232425262728293031323334 |  |
| --- | --- |
